# A sequential Monte Carlo approach to gene expression deconvolution

**DOI:** 10.1371/journal.pone.0186167

**Published:** 2017-10-19

**Authors:** Oyetunji E. Ogundijo, Xiaodong Wang

**Affiliations:** Department of Electrical Engineering, Columbia University, New York, New York, United States of America; Universitatsmedizin Greifswald, GERMANY

## Abstract

High-throughput gene expression data are often obtained from pure or complex (heterogeneous) biological samples. In the latter case, data obtained are a mixture of different cell types and the heterogeneity imposes some difficulties in the analysis of such data. In order to make conclusions on gene expresssion data obtained from heterogeneous samples, methods such as microdissection and flow cytometry have been employed to physically separate the constituting cell types. However, these manual approaches are time consuming when measuring the responses of multiple cell types simultaneously. In addition, exposed samples, on many occasions, end up being contaminated with external perturbations and this may result in an altered yield of molecular content. In this paper, we model the heterogeneous gene expression data using a Bayesian framework, treating the cell type proportions and the cell-type specific expressions as the parameters of the model. Specifically, we present a novel sequential Monte Carlo (SMC) sampler for estimating the model parameters by approximating their posterior distributions with a set of weighted samples. The SMC framework is a robust and efficient approach where we construct a sequence of artificial target (posterior) distributions on spaces of increasing dimensions which admit the distributions of interest as marginals. The proposed algorithm is evaluated on simulated datasets and publicly available real datasets, including Affymetrix oligonucleotide arrays and national center for biotechnology information (NCBI) gene expression omnibus (GEO), with varying number of cell types. The results obtained on all datasets show a superior performance with an improved accuracy in the estimation of cell type proportions and the cell-type specific expressions, and in addition, more accurate identification of differentially expressed genes when compared to other widely known methods for blind decomposition of heterogeneous gene expression data such as Dsection and the nonnegative matrix factorization (NMF) algorithms. MATLAB implementation of the proposed SMC algorithm is available to download at https://github.com/moyanre/smcgenedeconv.git.

## Introduction

Gene expression measurement technologies, for example, deoxyribonucleic acid (DNA) microarray, have made it possible to conduct simultaneous expression measurements from thousands of genes on a genome-wide scale [1–4]. Gene expression data obtained from pure samples, comprising of a single cell type, can be analyzed to yield a significant amount of information. For instance, measuring gene expression levels in different conditions may prove useful in medical diagnosis, treatment prescription, drug design [5, 6] and most importantly in the identification of genes that are differentially expressed between groups of samples [7], such as tumor versus non-tumor tissues [8].

However, in heterogeneous samples, where more than one cell types are present, drawing any reasonable conclusion is a difficult task because each of the cell types in the sample will contribute differently to the measured expression of a given gene [9]. In some cases, manual methods such as laser microdissection (LMD) [10] and flow cytometry [11] are employed to isolate cells of interest from the complex mixtures. In spite of that, there are some limitations in using these techniques. For instance, they are very expensive and often come with low cell throughput rate [12–14], resulting in a drastic reduction in the yield of biological contents.

In the literature, different computational methods have been proposed for the deconvolution of gene expression data from heterogeneous biological samples, and these methods can be loosely grouped into two categories: either deterministic or probabilistic. Of the two, the deterministic approach is more popular. For instance, in addition to the gene expression data, if the information about the cell-type specific gene expression profiles is available, proportions of cellular types can be estimated [15], for example, via linear regression [16–18], a very common technique for analyzing biological data [19]. On the other hand, if in addition to the gene expression data, cellular proportions are known, then with linear regression, cell-type specific gene expression profiles can be estimated [7, 20, 21]. Further, [22–24] investigated the efficacy of the nonnegative matrix factorization (NMF) algorithms [25, 26] for the “blind” deconvolution of gene expression data in the presence of additional constraints, for example, some prior biological knowledge [22, 23]. Moreover, [27] proposed a probabilistic approach based on the Markov chain Monte Carlo (MCMC) method, assuming an availability of a good initial estimate of the cell type proportions. All the approaches mentioned so far, either deterministic or probabilistic, made one or more assumptions about the availability, either precise or a rough estimate, of the cell type proportions or the cell-type specific profiles. But in reality, often times, all we have is the heterogeneous gene expression data.

In this paper, we propose a new probabilistic method, sequential Monte Carlo (SMC) sampler [28–31] for static models to estimate the cell type proportions and the cell-type specific expression profiles, given the heterogeneous gene expression data. Specifically, we model the heterogeneous gene expression data using a Bayesian framework where the cell-type specific expression profiles and the cell type proportions are the unknown model parameters. We seek to approximate, in an efficient way, the posterior distributions of all the unknown model parameters by a set of weighted samples (particles) from which their respective point estimates can be obtained. Bayesian inference is an important area in the analyses of biological data [32, 33] as it provides a complete picture of the uncertainty in the estimation of the unknown parameters of a model given the data and the prior distributions for all the unknown model parameters.

In particular, the SMC method is a class of sampling algorithms which combines importance sampling and resampling [34, 35]. More importantly, the SMC framework for static models is very similar to the sequential importance sampling (resampling) (SIS) procedure for dynamic models [34], the only difference being the framework under which the samples are propagated and this results in differences in the calculation of the weights of the samples. In general, SMC allows us to treat, in a principled way, any type of probability distribution, nonlinearity and non-stationarity [36, 37]. It is easy to implement and applicable to very general settings. As noted in [28], SMC algorithms address some of the major shortcomings of the MCMC-based algorithms: (i) diagnosing convergence of a Markov chain (ii) requirement of burn-in period, and (iii) MCMC algorithms getting trapped in local modes if the target distribution is highly multi-modal. In addition, in big data analyses, unlike the MCMC approach, SMC algorithms can be parallelized to reduce the computational time [28].

We compared the proposed SMC method with existing methods, including Dsection algorithm in [27] that is based on the MCMC approach and the recently proposed probabilistic nonnegative matrix factorization (PNMF) algorithm [38], a stochastic version of the deterministic NMF framework that takes into account the stochastic nature of the gene expression data. Overall, in terms of the accuracy of estimates of cell type proportions, cell-type specific gene expressions, and in addition, in the identification of differentially expressed genes, the proposed method demonstrated a superior performance. More importantly, the proposed method does not require that we have an initial estimate of the cell type proportions or the cell-type specific expression profiles.

The remainder of this paper is organized as follows. In Section 2, we present the Materials and Methods. In Section 3, we investigate the performance of the proposed method using simulated datasets artificially obtained from downloaded pure tissues expression profiles and heterogeneous (impure) samples downloaded from Affymetrix oligonucleotide arrays and GEO NCBI websites, the set of data that have been employed to assess the performance of deconvolution algorithms. Finally, Section 4 concludes the paper.

In this paper, we use the following notations:

1. *p*(⋅) and *p*(⋅|⋅) denote a probability and a conditional probability density functions, respectively.
2. $N(\mu ,\lambda^{-1})$ denotes the Gaussian probability density function with mean *μ*, precision *λ* and variance λ^−1^.
3. Gamma(*α*, *β*) denotes the Gamma probability density function with shape parameter *α* and rate parameter *β*.
4. U(a,b) denotes a uniform distribution with support *x* ∈ [*a*, *b*].
5. **x** and **x**^*T*^ denote a column vector and its transpose, respectively.
6. **X** and $\hat{X}$ denote a matrix and its estimate, respectively.

## Materials and methods

Let **Y** be an *I* × *J* gene expression matrix obtained from tissue samples with heterogeneous population, where *I* denotes the number of probes (or genes) in the measurements and *J* denotes the total number of samples present. We assume that the number of cell types, *K*, in the samples is known and each sample has the same number of cell types present, but in varying percentages. Although, modeling the relationship between the expression value of pure and mixed samples is not strictly linear, linearity has proved to be a reasonable and valid assumption in gene expression deconvolution [7, 16, 27, 39]. As such, we follow the linear modeling approach in analyzing the tissue samples. Denoting the indices of cell type, tissue sample and gene by *k*, *j* and *i*, respectively, then the expression value of gene *i* in sample *j* is the sum of its expressions in all *K* cell types, i.e.,

$$\begin{array}{c} \begin{array}{c} y_{ij}=\sum_{k=1}^{K}x_{ik}m_{kj}+e_{ij},\;i=1,\ldots ,I,\;j=1,\ldots ,J,\; \end{array} \end{array}$$ (1)

where *x*_*ik*_ denotes the specific expression of gene *i* in cell type *k*, *m*_*kj*_ denotes the proportion of cell type *k* in sample *j* and *e*_*ij*_ is an additive Gaussian distributed noise with zero mean and precision λ (inverse of variance). Instead of one gene at a time, if all the genes are considered at once, then (1) can be written in a matrix form as follows:

$$\begin{array}{c} Y=XM+E, \end{array}$$ (2)

where **Y** denotes the *I* × *J* matrix of gene expression measurement from heterogeneous samples, **X** denotes the unknown *I* × *K* matrix of expression levels of the genes in all the cell types (pure cell type expression signatures), **M** denotes the unknown *K* × *J* matrix of cell type proportions and **E** is the additive noise matrix of dimension *I* × *J*. Note that all elements of **M** are non-negative and each column sums to 1.

The goal of the inference is to obtain an estimate of the unknown matrices **X** and **M**, which are the cell-type specific signatures and the cellular proportions, respectively and in addition, an estimate of the precision λ, given the heterogeneous gene expression matrix **Y**. To do this, we define a data generating model, impose prior distributions on all the unknown model parameter, derive the sequence of target distributions for all the model parameters and finally, present the SMC algorithm that estimates, in an efficient manner, the posterior distributions of all the unknown model parameters.

### Likelihood function

As shown in (1), the data point for probe *i* in sample *j* i.e., *y*_*ij*_, is modeled as a sum of the cell-type specific expressions of probe *i* for all cell types, i.e. the *i*^*th*^ row of matrix **X**, denoted by **x**_*i*,:_, weighted by the proportions of all cell types in sample *j*, i.e., the *j*^*th*^ column of matrix **M**, denoted by **m**_:,*j*_ plus an additive Gaussian distributed noise, *e*_*ij*_ i.e.,

$$\begin{array}{c} \begin{array}{c} p\left(y_{ij}|x_{i,:},m_{:,j},\lambda \right)=N\left(x_{i,:}m_{:,j},\lambda^{-1}\right)\;=N(\sum_{k=1}^{K}x_{ik}m_{kj},\lambda^{-1}). \end{array} \end{array}$$ (3)

Further, if we assume independent and identically distributed (IID) measurements for the data points in matrix **Y**, then the joint data likelihood function can be written as:

$$\begin{array}{c} \begin{array}{c} p\left(Y|\theta \right)=\prod_{i=1}^{I}\prod_{j=1}^{J}p\left(y_{ij}|x_{i,:},m_{:,j},\lambda \right), \end{array} \end{array}$$ (4)

where ***θ*** = {λ, *x*_*ik*_, *m*_*kj*_: *i* = 1, …, *I*, *j* = 1, …, *J*, *k* = 1, …, *K*} are the unknown parameters of the model that will be estimated.

### Prior densities for all model parameters

Here, we present the prior distributions for all the unknown parameters in the model in (4). With the prior distributions accurately specified and with the model in (4), we can obtain the sequence of target distributions for all the unknown model parameters.

#### Prior densities for the cell-type specific expressions

We model the specific expression of gene *i* in cell type *k*, *x*_*ik*_ with a Gaussian distribution, i.e., $x_{ik}∼N\left(\mu_{ik},\nu_{ik}^{-1}\right)$, where *μ*_*ik*_ and *ν*_*ik*_ are the mean and precision, respectively, and are assumed known [27, 38]. Gaussian distribution is preferred so as to make use of the property of conjugate priors, i.e., the sequence of target distributions will remain Gaussian given that the prior and the likelihood distributions are Gaussian [40]. Detailed derivations of the sequence of target distributions and the choice of *μ*_*ik*_ and *ν*_*ik*_ are discussed in S1 Supplementary Material.

#### Prior densities for the cell type proportions

We impose a Gaussian distribution on the proportion of cell type *k* in sample *j*, *m*_*kj*_ i.e, $m_{kj}∼N\left(\mu_{kj},\nu_{kj}^{-1}\right)$, where *μ*_*kj*_ and *ν*_*kj*_ are the mean and precision, respectively, and are assumed known [38]. Although, other distributions can be considered, surprisingly, Gaussian distribution performs well in our experiments. Detailed derivations of the sequence of target distributions and the how *μ*_*kj*_ and *ν*_*kj*_ are picked are discussed in S1 Supplementary Material.

#### Prior density for the precision

Gamma prior is placed on the inverse of the noise variance (precision), i.e, λ ∼ Gamma(*α*, *β*), with *α* and *β* assumed known. The choice of Gamma prior distribution ensures that the sequence of target distributions for the precision parameter will be Gamma distributions (conjugate prior property), given that the likelihood is a Gaussian distribution [40]. Detailed derivations of the sequence of target distributions and the choice of *α* and *β* are discussed in S1 Supplementary Material.

### Sequential Monte Carlo samplers for Bayesian inference

#### General principle of SMC samplers

Before we introduce the SMC sampler algorithm for gene expression decomposition, we will succinctly describe the general principle of SMC samplers in Bayesian inference settings [28–30]. Denote the prior distribution, the likelihood function and the posterior distribution in a Bayesian inference setup as *p*(***θ***), *p*(**Y**|***θ***) and *p*(***θ***|**Y**), respectively. Using the Bayes rule, the posterior distribution can be written as a function of the prior distribution and the likelihood function as follows:

$$\begin{array}{c} \begin{array}{c} p\left(\theta |Y\right)=\frac{p(\theta )p(Y|\theta )}{Z} \end{array} \end{array}$$ (5)

where **Z** = ∫_Θ_ *p*(***θ***)*p*(**Y**|***θ***)*d****θ***, a constant with respect to ***θ***, is referred to as the evidence. With SMC samplers, rather than sampling from the posterior distribution *p*(***θ***|**Y**) in (5), a sequence of intermediate target distributions, ${\left\{\pi_{t}\right\}}_{t=1}^{T}$, are designed, that transitions smoothly from the prior distribution, i.e., *π*_1_ = *p*(***θ***), which is usually easier to sample from, and gradually introduce the effect of the likelihood so that in the end, we have *π*_*T*_ = *p*(***θ***|**Y**) which is the posterior distribution of interest [28, 29]. For such sequence of intermediate distributions, a natural choice is the likelihood tempered target sequence [28, 41]:

$$\begin{array}{c} \begin{array}{c} \pi_{t}\left(\theta \right)=\frac{\Psi_{t}\left(\theta \right)}{Z_{t}}\propto p\left(\theta \right)p{\left(Y|\theta \right)}^{\epsilon_{t}}, \end{array} \end{array}$$ (6)

where ${\left\{\epsilon_{t}\right\}}_{t=1}^{T}$ is a non-decreasing temperature schedule with *ϵ*_1_ = 0 and *ϵ*_*T*_ = 1, $\Psi_{t}\left(\theta \right)=p\left(\theta \right)p{\left(\theta |Y\right)}^{\epsilon_{t}}$ is the unnormalized target distribution and $Z_{t}=\int_{\Theta }p\left(\theta \right)p{\left(\theta |Y\right)}^{\epsilon_{t}}d\theta$ is the evidence at time *t*.

Next, we transform this problem in the standard SMC filtering framework [34, 35] by defining a sequence of joint target distributions up to and including time *t*, ${\left\{{\tilde{\pi }}_{t}\right\}}_{t=1}^{T}$ which admits *π*_*t*_ as marginals as follows:

$$\begin{array}{c} \begin{array}{c} {\tilde{\pi }}_{t}\left(\theta_{1:t}\right)=\frac{{\tilde{\Psi }}_{t}\left(\theta_{1:t}\right)}{Z_{t}},\;\text{with}\;{\tilde{\Psi }}_{t}\left(\theta_{1:t}\right)=\Psi_{t}\left(\theta_{t}\right)\prod_{b=1}^{t-1}L_{b}\left(\theta_{b+1},\theta_{b}\right), \end{array} \end{array}$$ (7)

where the artificial kernels ${\left\{L_{b}\right\}}_{b=1}^{t-1}$ are referred to as the backward Markov kernels, i.e., $L_{t}\left(\theta_{t+1},\theta_{t}\right)$ denotes the probability density of moving back from ***θ***_*t*+1_ to ***θ***_*t*_ [28, 29, 42]. However, it is often difficult to sample directly from the joint target distribution in (7). Instead, samples are obtained from another distribution, known as the importance distribution, with a support that includes the support of ${\tilde{\pi }}_{t}$ [34]. Thus, we define the importance distribution at time *t*, *q*_*t*_(***θ***_1:*t*_) as follows:

$$\begin{array}{c} \begin{array}{c} q_{t}\left(\theta_{1:t}\right)=q_{1}\left(\theta_{1}\right)\prod_{f=2}^{t}K_{f}\left(\theta_{f-1},\theta_{f}\right), \end{array} \end{array}$$ (8)

where ${\left\{K_{f}\right\}}_{f=2}^{t}$ are the Markov transition kernels or forward kernels, i.e., $K_{t}\left(\theta_{t-1},\theta_{t}\right)$ denotes the probability density of moving from ***θ***_*t*−1_ to ***θ***_*t*_ [28, 29].

Given that at time *t* − 1, we desire to obtain *N* random samples from the target distribution in (7), but as discussed earlier, it is difficult to sample from the target distribution and instead, we obtain the samples from the importance distribution in (8). Following the principle of importance sampling, we then correct for the discrepancy between the target and the importance distributions by calculating the importance weights [34]. The unnormalized weights associated with the *N* samples are obtained as follows:

$${\tilde{w}}_{t-1}^{n}\propto \frac{{\tilde{\pi }}_{t-1}(\theta_{1:t-1}^{n})}{q_{t-1}(\theta_{1:t-1}^{n})}=\frac{\pi_{t-1}(\theta_{t-1}^{n})\prod_{d=1}^{t-2}L_{d}(\theta_{d+1}^{n},\theta_{d}^{n})}{q_{1}(\theta_{1}^{n})\prod_{r=2}^{t-1}K_{r}(\phi_{r-1}^{n},\theta_{r}^{n})}$$ (9)

and the normalized weights are calculated as:

$$w_{t-1}^{n}=\frac{{\tilde{w}}_{t-1}^{n}}{\sum_{l=1}^{N}{\tilde{w}}_{t-1}^{l}},n=1,\ldots ,N.$$

As such, the set of weighted samples ${\left\{\theta_{1:t-1}^{n},w_{t-1}^{n}\right\}}_{n=1}^{N}$ approximates the joint target distribution ${\tilde{\pi }}_{t-1}$. To obtain an approximation to the joint target distribution at time *t*, i.e, ${\tilde{\pi }}_{t}$, the samples are first propagated to the next target distribution ${\tilde{\pi }}_{t}$ using a forward Markov kernel $K_{t}\left(\theta_{t-1},\theta_{t}\right)$ to obtain the set of particles ${\left\{\theta_{1:t}^{n}\right\}}_{n=1}^{N}$. Similar to (9), we then correct for the discrepancy between the importance distribution and the target distribution at time *t*. Thus, the unnormalized weights at time *t* are calculated as follows:

$$\begin{array}{ll} {\tilde{w}}_{t}^{n} & \propto \frac{{\tilde{\pi }}_{t}(\theta_{1:t}^{n})}{q_{t}(\theta_{1:t}^{n})}\\ & =\frac{\pi_{t}(\theta_{t}^{n})\prod_{d=1}^{t-1}L_{d}(\theta_{d+1}^{n},\theta_{d}^{n})}{q_{1}(\theta_{1}^{n})\prod_{r=2}^{t}K_{r}(\theta_{r-1}^{n},\theta_{r}^{n})}\\ & =\frac{\pi_{t}(\theta_{t}^{n})L_{t-1}(\theta_{t}^{n},\theta_{t-1}^{n})\prod_{d=1}^{t-2}L_{d}(\theta_{d+1}^{n},\theta_{d}^{n})}{q_{1}(\theta_{1}^{n})K_{t}(\theta_{t-1}^{n},\theta_{t}^{n})\prod_{r=2}^{t-1}K_{r}(\theta_{r-1}^{n},\theta_{r}^{n})}\\ & =\frac{\pi_{t}(\theta_{t}^{n})L_{t-1}(\theta_{t}^{n},\theta_{t-1}^{n})\pi_{t-1}(\theta_{t-1}^{n})\prod_{d=1}^{t-2}L_{d}(\theta_{d+1}^{n},\theta_{d}^{n})}{\pi_{t-1}(\theta_{t-1}^{n})K_{t}(\theta_{t-1}^{n},\theta_{t}^{n})q_{1}(\theta_{1}^{n})\prod_{r=2}^{t-1}K_{r}(\theta_{r-1}^{n},\theta_{r}^{n})} \end{array}$$ (10)

from (9), we have

$${\tilde{w}}_{t}^{n}\propto {\tilde{w}}_{t-1}^{n}\frac{\pi_{t}(\theta_{t}^{n})L_{t-1}(\theta_{t}^{n},\theta_{t-1}^{n})}{\pi_{t-1}(\theta_{t-1}^{n})K_{t}(\theta_{t-1}^{n},\theta_{t}^{n})},$$

from the definitions of *π*_*t*_ and *π*_*t*−1_ in (6) and noticing that **Z**_*t*_ and **Z**_*t*−1_ are constants with respect to $\theta_{t}^{n}$ and $\theta_{t-1}^{n}$, then

$$\begin{array}{cc} {\tilde{w}}_{t}^{n} & \propto {\tilde{w}}_{t-1}^{n}\frac{\Psi_{t}(\theta_{t}^{n})L_{t-1}(\theta_{t}^{n},\theta_{t-1}^{n})}{\Psi_{t-1}(\theta_{t-1}^{n})K_{t}(\theta_{t-1}^{n},\theta_{t}^{n})}\\ & ={\tilde{w}}_{t-1}^{n}W_{t}(\theta_{t-1}^{n},\theta_{t}^{n}),n=1,\ldots ,N, \end{array}$$

where ${\left\{{\tilde{w}}_{t-1}^{n}\right\}}_{n=1}^{N}$ are the unnormalized weights at time *t* − 1, given in (9) and ${\left\{W_{t}\left(\theta_{t-1}^{n},\theta_{t}^{n}\right)\right\}}_{n=1}^{N}$, the unnormalized incremental weights, calculated as

$$\begin{array}{c} \begin{array}{c} W_{t}\left(\theta_{t-1}^{n},\theta_{t}^{n}\right)=\frac{\Psi_{t}\left(\theta_{t}^{n}\right)L_{t-1}\left(\theta_{t}^{n},\theta_{t-1}^{n}\right)}{\Psi_{t-1}\left(\theta_{t-1}^{n}\right)K_{t}\left(\theta_{t-1}^{n},\theta_{t}^{n}\right)},\;n=1,\ldots ,N. \end{array} \end{array}$$ (11)

#### Resampling procedure

In the SMC procedure described above, after some iterations, all samples except one will have very small weights, a phenomenon referred to as degeneracy in the literature. It is unavoidable as it has been shown that the variance of the importance weights increases over time [34]. An adaptive way to check this is by computing the effective sample size (ESS) as follows: $ESS=1/\Sigma_{n=1}^{N}{\left(w_{t}^{n}\right)}^{2}$ [43]. To avoid degeneracy, one performs resampling when the *ESS* is significantly less than the number of samples, discarding the ineffective samples and then multiply the effective ones [37, 44]. In all our experiments, we performed resampling when the *ESS* is less than *N*/10 [45]. The resampling procedure is briefly summarized as follows:

- Interpret each weight $w_{t}^{n}$ as the probability of obtaining the sample index *n* in the set $\left\{\theta_{t}^{n}:n=1,\ldots ,N\right\}$.
- Draw *N* samples from the discrete probability distribution and replace the old sample set with this new one.
- Set all weights to the constant value $w_{k}^{n}=1/N$.

#### Target distributions, forward and backward kernels specification for gene expression deconvolution

In (6)–(8), we need to specify the exact form of the sequence of target distributions ${\left\{\pi_{t}\right\}}_{t=1}^{T}$, the forward kernels, ${\left\{K_{t}\right\}}_{t=2}^{T}$ and the backward kernels ${\left\{L_{t-1}\right\}}_{t=2}^{T}$ for the problem of gene expression deconvolution.

- Sequence of target distributions and forward kernels: As earlier discussed, we are interested in the likelihood tempered target sequence in (6). Here, we present the sequence of target distributions for all the parameters in the model presented in (4). Details of the derivations are in S1 Supplementary Material. Define $Y_{ijk}=\Sigma_{k^{\prime }\neq k}x_{ik^{\prime }}m_{k^{\prime }j},$ then the *sequence of target distributions for the cell type proportions* are:
  $$\begin{array}{c} \begin{array}{cc} & \pi_{t}\left(m_{kj}|\cdot \right)=N(\frac{V_{kj}^{t}}{U_{kj}^{t}},\frac{1}{U_{kj}^{t}}),\text{where}\;U_{kj}^{t}=\nu_{kj}+\epsilon_{t}\lambda \sum_{i=1}^{I}x_{ik}^{2},\\ & \;V_{kj}^{t}=\mu_{kj}\nu_{kj}+\epsilon_{t}\lambda (\sum_{i=1}^{I}y_{ij}x_{ik}-\sum_{i=1}^{I}Y_{ijk}x_{ik}),k=1,\ldots ,K,\;j=1,\ldots ,J,\;t=1,\ldots ,T, \end{array} \end{array}$$ (12)
  the *sequence of target distributions for the cell-type specific expressions* are given as:
  $$\begin{array}{c} \begin{array}{cc} & \pi_{t}\left(x_{ik}|\cdot \right)=N(\frac{B_{ik}^{t}}{A_{ik}^{t}},\frac{1}{A_{ik}^{t}}),\text{where}\;A_{ik}^{t}=\nu_{ik}+\epsilon_{t}\lambda \sum_{j=1}^{J}m_{kj}^{2},\\ & \;B_{ik}^{t}=\mu_{ik}\nu_{ik}+\epsilon_{t}\lambda (\sum_{j=1}^{J}y_{ij}m_{kj}-\sum_{j=1}^{J}Y_{ijk}m_{kj}),i=1,\ldots ,I,\;k=1,\ldots ,K,\;t=1,\ldots ,T, \end{array} \end{array}$$ (13)
  and finally, the *sequence of target distributions for the precision* are given as:
  $$\begin{array}{l} \pi_{t}(\lambda |\cdot )=\text{Gamma}(\tilde{\alpha },\tilde{\beta }),\text{where}\;\tilde{\alpha }=\alpha +\frac{\epsilon_{t}IJ}{2}\;\text{and}\\ \tilde{\beta }=\beta +\frac{\epsilon_{t}}{2}\sum_{i=1}^{I}\sum_{j=1}^{J}(y_{ij}-\sum_{k=1}^{K}x_{ik}m_{kj}{)}^{2},t=1,\ldots ,T. \end{array}$$ (14)
  The optimal forward Markov kernel, in the sense of minimizing the variance of the importance weights is $K_{t}\left(\theta_{t-1},\theta_{t}\right)=\pi_{t}\left(\theta_{t}\right)$ [28, 29]. In general, if *π*_*t*_ is not available in closed form (non-conjugate priors), then an MCMC kernel of invariant distribution *π*_*t*_ will be used for $K_{t}$ (Metropolis-Hastings MCMC). Fortunately, in our model, we are able to compute the sequence ${\left\{\pi_{t}\right\}}_{t=1}^{T}$ analytically as shown in (12)–(14).
- Sequence of backward kernels: In order to obtain a good performance, the backward kernel is optimized with respect to the forward kernel as this choice will affect the variance of the importance weights. Hence, the following $L_{t}$ is employed [28, 30]:
  $$\begin{array}{c} \begin{array}{c} L_{t-1}\left(\theta_{t},\theta_{t-1}\right)=\frac{\pi_{t}\left(\theta_{t-1}\right)K_{t}\left(\theta_{t},\theta_{t-1}\right)}{\pi_{t}\left(\theta_{t}\right)}, \end{array} \end{array}$$ (15)
  since it generally represents a good approximation of the optimal backward kernel when the discrepancy between *π*_*t*_ and *π*_*t*−1_ is small [29, 31]. Thus, the unnormalized incremental weights in (11) become:
  $$\begin{array}{c} \begin{array}{cc} W_{t}\left(\theta_{t-1}^{n},\theta_{t}^{n}\right) & =\frac{\Psi_{t}\left(\theta_{t}^{n}\right)\pi_{t}\left(\theta_{t-1}^{n}\right)}{\Psi_{t-1}\left(\theta_{t-1}^{n}\right)\pi_{t}\left(\theta_{t}^{n}\right)}\\ & =\frac{p\left(\theta_{t}^{n}\right)p{\left(Y|\theta_{t}^{n}\right)}^{\epsilon_{t}}p\left(\theta_{t-1}^{n}\right)p{\left(Y|\theta_{t-1}^{n}\right)}^{\epsilon_{t}}}{p\left(\theta_{t-1}^{n}\right)p{\left(Y|\theta_{t-1}^{n}\right)}^{\epsilon_{t-1}}p\left(\theta_{t}^{n}\right)p{\left(Y|\theta_{t}^{n}\right)}^{\epsilon_{t}}}\\ & =p{\left(Y|\theta_{t-1}^{n}\right)}^{\left(\epsilon_{t}-\epsilon_{t-1}\right)},\;n=1,\ldots ,N, \end{array} \end{array}$$ (16)
  where *ϵ*_*t*_ − *ϵ*_*t*−1_ is the step length of the cooling schedule of the likelihood at time *t*. The derivation of the exact analytical expression in (16) for the gene expression deconvolution problem is presented in S1 Supplementary Material.

Finally, since the unnormalized incremental weights in (16) at time *t* does not depend on the particle values at time *t* but just on the previous particle set, the particles ${\left\{\theta_{t}^{n}\right\}}_{n=1}^{N}$ should be sampled after the weights ${\left\{{\tilde{w}}_{t}^{n}\right\}}_{n=1}^{N}$ have been computed and after the particle approximation $\left\{{\tilde{w}}_{t}^{n},\theta_{t-1}^{n}\right\}$ has possibly been resampled [28].

### SMC sampler algorithm for gene expression deconvolution

1. Input: Heterogeneous gene expression matrix **Y**, *α*, *β*, {*μ*_*kj*_, *ν*_*kj*_: *k* = 1, …, *K*, *j* = 1, …, *J*}, {*μ*_*ik*_, *ν*_*ik*_: *i* = 1, …, *I*, *k* = 1, …, *K*}, and the temperature schedule 0 = *ϵ*_1_ < *ϵ*_2_…<*ϵ*_*T*_ = 1 (See the S1 Supplementary Material for the initial values).

2. Set *t* = 1

 for *n* = 1: *N*

  Take a sample from Gamma(*α*, *β*).

  for *k* = 1: *K*

   for *j* = 1: *J*

    Take a sample from $N(\mu_{kj},\nu_{kj}^{-1})$.

   end

  end

  for *i* = 1: *I*

   for *k* = 1: *K*

    Take a sample from $N(\mu_{ik},\nu_{ik}^{-1})$.

   end

  end

 end

 Set $w_{1}^{n}=1/N,\;n=1,\ldots ,N$.

3. for *t* = 2: *T* repeat the following steps:

 • Compute the unnormalized weights as follows using (16):

$${\tilde{w}}_{t}^{n}=w_{t-1}^{n}p{\left(Y|\theta_{t-1}\right)}^{\left(\epsilon_{t}-\epsilon_{t-1}\right)},\;n=1,\ldots ,N.$$

 • Normalization of the weights:

$$w_{t}^{n}=\frac{{\tilde{w}}_{t}^{n}}{\sum_{l=1}^{N}{\tilde{w}}_{t}^{l}},\;n=1,\ldots ,N.$$

 • Compute $ESS=1/\Sigma_{n=1}^{N}{\left(w_{t}^{n}\right)}^{2}$ and resample if *ESS* < *N*/10.

 • Propagation of particles:

  for *n* = 1: *N*

   Take a sample from *π*_*t*_(λ|⋅) in (14).

   for *k* = 1: *K*

    for *j* = 1: *J*

     Take a sample from *π*_*t*_(*m*_*kj*_|⋅) in (12).

    end

   end

   for *i* = 1: *I*

    for *k* = 1: *K*

     Take a sample from *π*_*t*_(*x*_*ik*_|⋅) in (13).

    end

   end

  end

 end

4. Compute the estimate of the parameters as follows:

$$\begin{array}{c} \hat{\theta }=\sum_{n=1}^{N}w_{T}^{n}\theta_{T}^{n}, \end{array}$$ (17)

then the estimates of the cell type proportions matrix $\hat{M}$, cell-type specific expression matrix $\hat{X}$ and the precision $\hat{\lambda }$ are obtained from $\hat{\theta }$ for further analyses (Note that each column of $\hat{M}$ is re-scaled to sum to unity).

## Results

### Ground-truth for variables

We assessed the performance of the proposed method, which we will refer to as the SMC method, on both simulated dataset and datasets that contain real mixed samples. For ease of exposition, denote $Y_{total}=\left[Y,\tilde{Y}\right]$, where matrix **Y**_*total*_ is the downloaded matrix of pure and mixed gene expressions, matrix **Y** is the gene expression for the heterogeneous/mixed samples and $\tilde{Y}$ is the gene expression matrix for the pure samples (the expression profile of each sample often come in multiplicity, e.g., technical replicates). First, we compared the estimates of the cell types proportion and the cell-type specific expression matrices with some existing methods and secondly, we went further to test the ability of the proposed method to identify differentially expressed genes. Next, we present the “ground-truth” for all the unknown variables in our analyses. Unless otherwise stated, all the datasets used in the analyses are not log transformed.

#### Ground-truth for the cell types proportions and the cell-type specific expression profiles (matrices M and X)

For all datasets, “ground-truth” is available for the cell type proportions matrix **M**. For the pure cell-type expression signatures, matrix **X**, “ground-truth” is computed from the matrix $\tilde{Y}$, the gene expression for the pure samples. Denote $\tilde{Y}=\left[{\tilde{Y}}^{1},{\tilde{Y}}^{2},\ldots ,{\tilde{Y}}^{K}\right]$, where ${\tilde{Y}}^{k},k\in \left\{1,\ldots ,K\right\}$, is the gene expression matrix that contains replicate samples from pure cell type *k*, then, *x*_*ik*_ is computed as the mean of row *i* in matrix ${\tilde{Y}}^{k}$, that is, the mean expression for gene *i* across samples that contain only cell type *k*.

#### List of differentially and non-differentially expressed genes

We produced the “ground-truth” for the list of differentially expressed and non-differentially expressed genes from the “ground-truth” for the cell-type expression signatures, matrix **X**, using the fold change rule (Although, the median fold change proposed in [46] is theoretically a slightly better alternative to the mean fold change, empirical results from both method are similar for all our datasets. More so, mean fold change is better suited to our purpose because in the end, we estimate the mean expression for each cell type [47]). For gene *i*, the fold change between cell types *r* and *u* is defined as: *FC*_*i*_ = max(*x*_*ir*_, *x*_*iu*_)/min(*x*_*ir*_, *x*_*iu*_), where *x*_*ir*_ and *x*_*iu*_ are the specific expressions of gene *i* in cell types *r* and *u*, *r*, *u* ∈ {1, …, *K*} [46–48]. Thus, given the specific expressions of gene *i* in cell types *r*, *u* ∈ {1, …, *K*}, if *FC*_*i*_ > 2, gene *i* is said to be differentially expressed in the two cell types, otherwise no difference in expressions [49].

#### Cell types mapping and marker probesets

Estimates of the cell-type specific expression profiles obtained from any blind decomposition algorithm require mapping to the correct cell types [22]. As such, marker probesets are often employed to perform the mapping of the estimated profiles to the true cell types. However, gene expression data are generated with different technologies (microarrays and RNA-seq) using equipment from different manufacturers (e.g. Affymetrix, Illumina etc.). To avoid discrepancies that may arise in using probeset marker lists from another source due to probe annotation [50, 51], we defined the list of marker probesets used in our experiments from the gene expression measurements of pure cell types/tissues samples, i.e. matrix $\tilde{Y}$ and matrix **X**, following the procedures highlighted in [22]. Details of how the marker probesets are defined and the mapping of the estimated profiles to the true cell types are discussed in S1 Supplementary Material.

#### Metrics for comparing results

Notice that the mapping of estimated cell-type profiles to the true cell types also rearranges the rows of the estimated proportions, matrix $\hat{M}$. Now, to compare the estimated variables with the true values, we compared the average mean absolute difference for the simulated datasets and then calculated the Pearson correlation coefficient (*r*) between the true value and the estimated value for the real data.

In addition, we tested if the proposed SMC method can identify differentially expressed genes between cell types. Given the “ground-truth” for the truly differentially and non-differentially expressed genes, we computed, for each probeset, the expression fold change between the columns of the estimated cell-type gene expression profiles, matrix $\hat{X}$. Specifically, between any two columns of matrix $\hat{X}$ and for each probeset (and if cell type 1 is upregulated when compared to cell type 2 or vice-versa, separately), we computed the following by varying the fold change threshold from 1 to 5 in step of 0.25: true positives (TP), the number of correctly identified probes that are truly differentially expressed; false positives (FP), the number of non-differentially expressed probes but incorrectly identified as differentially expressed genes; false negatives (FN), the number of truly differentially expressed genes but incorrectly identified as non-differentially expressed probes, and true negatives (TN), the number of correctly identified non-differentially expressed probes. Further, we computed the sensitivity or true positive rate (TPR) = TP/(TP+FN) and the false positive rate (FPR), also defined as 1− specificity = FP/(FP+TN). With the TPR and the FPR for the different threshold values, we generated the receiver operating characteristic curves (ROC) for all pairs of cell types. Area under the ROC (AUROC) is obtained for each plot. High value of AUROC (maximum is 1) indicates that the deconvolution method is specific and sensitive in identifying differentially expressed probeset.

In addition, to compare our method with other existing gene expression deconvolution methods that require same set of input data, we analyzed the datasets with two other methods: another sampling algorithm developed by [27] which we will refer to as the MCMC method and a recently developed probabilistic version of NMF [38] which we will refer to as the PNMF method. Although, the MCMC method assumes that a rough estimate of the mixing proportions might be available, in some cases, in addition to the gene expression data, we initialized all methods with equal cell type proportion in order to produce a fair comparison of the results. Also, for the NMF method, cell-type specific gene expression profiles, matrix **X** is initialized by drawing its entries from a uniform distribution U(0,max(Y)).

### Simulated dataset

To test the proposed algorithm on simulated data, we created heterogeneous gene expression datasets with varying number of samples from pure tissue samples. Specifically, we downloaded the gene expression measurements (tissue specific gene expression data) from the publicly available dataset series GSE1133, from the GEO website [52] for human lung, heart and liver. Data preprocessing, that is, background adjustment, normalization, and summarization were done with robust multi-array average (RMA) procedure [53]. For the cell type proportion matrix **M**, each column of the matrix is generated from a Dirichlet distribution. Heterogeneous gene expression measurement is then created by multiplying the tissue specific gene expression profiles, matrix **X** by the simulated cell type proportions, matrix **M**. Finally, normally distributed noise with mean zero and variance that is equal to the global variance in gene expression between duplicate samples in GSE1133, is added. Then, we created heterogeneous gene expression data, matrix **Y** that comprises of 10, 15, 20, 25, 30, 35 and 40 samples, respectively.

**Fig 1:**
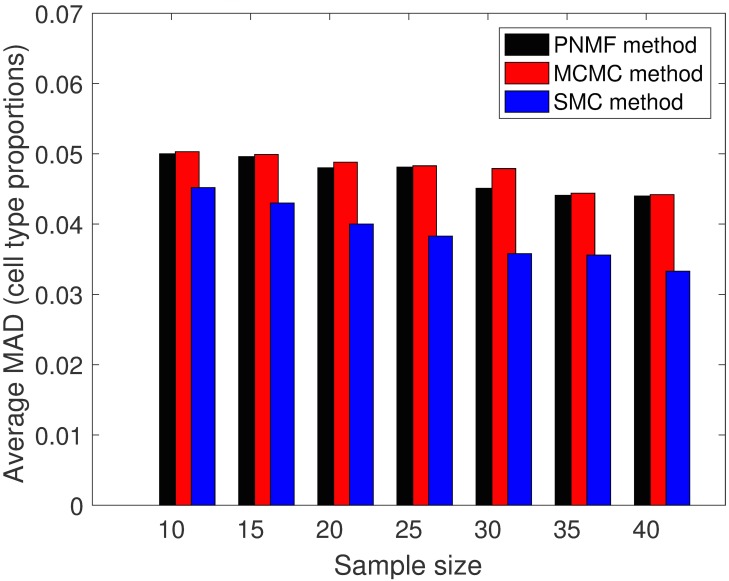
Plot of average MAD for different sample size. Plot of average MAD calculated from varying the sample size for all the methods (simulated datasets).

**Fig 2:**
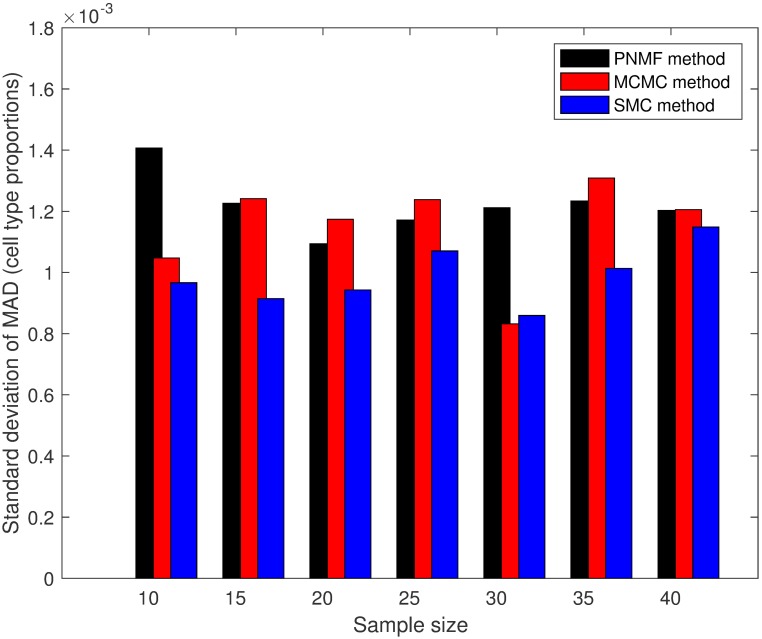
Plot of standard deviation of MAD. Plot of standard deviation of MAD for all the methods (simulated datasets).

**Fig 3:**
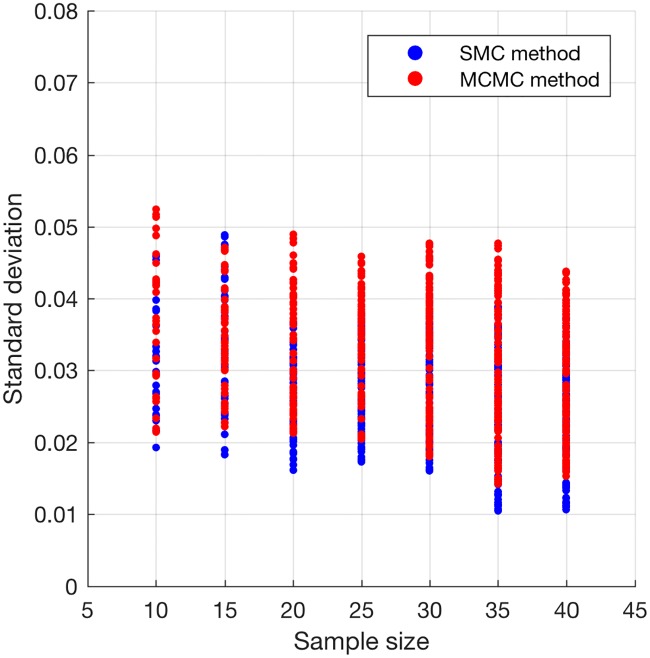
Plot of standard deviation of parameter estimates. Standard deviation of the estimates obtained from the proposed SMC and MCMC methods.

With each sample size, we made 25 experimental runs with each of the proposed SMC algorithm, MCMC method and the PNMF method. For each of the methods and a sample size, we record the mean absolute difference (MAD) between the true cell type proportions and the estimated cell type proportions after each experimental run and average MAD was computed after 25 runs. The results for the average and the standard deviation of MAD for the three methods and all the sample sizes are presented in Figs 1 and 2. In addition, for each sample size, we took the average of the estimated standard deviations over the 25 experimental runs. For each sample size, we showed, in Fig 3, a scatter plot of the standard deviations for the SMC and the MCMC methods (PNMF algorithm returned only the maximum a posteriori (MAP) estimates). Overall, the proposed SMC method outperforms its two other counterparts across all the sample sizes, in terms of the accuracy of the estimates. In addition, it can be seen that as the number of sample sizes goes up, estimates of model parameters also improve.

Moreover, we investigated how much the results obtained from the proposed SMC algorithm depends on the choice of the prior distributions. Specifically, we considered a Dirichlet distribution for modeling each column of the cell type proportions (non-conjugate prior), matrix **M**. With this choice of prior distribution, the sequence of target distributions *π*_*t*_ for the mixture proportions are no more in closed form as we have in (12). Thus, to propagate the particles after the resampling procedure in the proposed SMC algorithm, we employed an Metropolis-Hastings MCMC kernel of invariant distribution *π*_*t*_ [28]. For each particle, we ran 10 chains and the last iteration is chosen as the propagated particle. On the GSE1133 dataset with 10 samples and 500 randomly chosen genes, the results obtained for the conjugate and the non-conjugate prior distributions (Dirichlet distributions) are shown in Table 1. Particularly, we recorded the correlation coefficient (*r*) and the runtime for the two cases on a 3.5 Ghz Intel 8 processors running MATLAB. From Table 1, the two cases yielded similar results in terms of the accuracy of the estimates, but the algorithm implemented with the non-conjugate priors is slower than its counterpart with conjugate priors. This is due to the fact that the MCMC kernel used in propagating the particles ran multiple iterations for each particle, and the similarity in the results is because the MCMC kernel used has an invariant distribution *π*_*t*_, where the particles are sampled from.

**Table 1:** Effect of the choice of priors for the proposed SMC algorithm.

	SMC with conjugate priors	SMC with non-conjugate priors
*r*	0.99	0.99
Runtime (minutes)	132	226

Lastly, on the same dataset, we performed experiments with the MCMC method and the PNMF algorithm. In particular, the MCMC was run with chain length of 40,000, with the initial 20000 as burn-in and a thinning interval of 20. The results are shown in Table 2

**Table 2:** Runtime of different methods on the same dataset.

	SMC method	MCMC method	PNMF method
Runtime (minutes)	132	116	84
*r*	0.99	0.93	0.95

### Affymetrix dataset: 2 cell types

Next, we evaluated the performance of the proposed SMC algorithm on a tissue mixture oligonucleotide microarray probe-level dataset from Affymetrix previously analyzed by [27]. Data preprocessing were done by the RMA procedure [53]. This dataset, **Y**_*total*_, consists of heterogeneous expressions from human brain and heart cells. There are 33 samples and each sample comprises of specific proportions of the two distinct cell types. The true mixture proportions are shown in Table A in S1 Supplementary Material where the samples are designated S1,…,S33 for sample 1,…,sample 33, respectively. Samples S1—S3 and S31—S33, samples from the pure cell types, constitute the matrix $\tilde{Y}$, for approximating the “ground-truths” for the cell-type expression profiles (matrix **X**), marker probesets and the list of truly differentially expressed and non-differentially expressed genes. Samples S4—S30 constitute the heterogeneous gene expression matrix **Y** that was analyzed.

**Fig 4:**
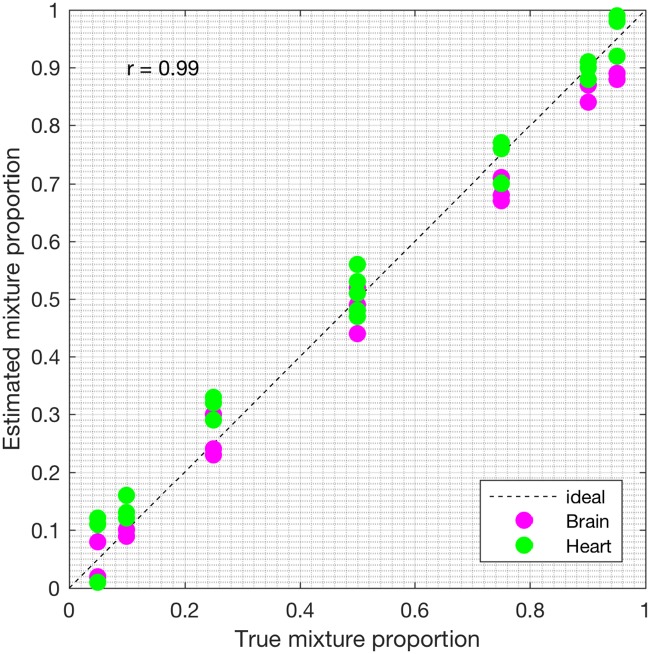
Plot of proportions. Plot of the true proportions vs. estimated proportions obtained from the proposed SMC method (affymetrix dataset).

**Fig 5:**
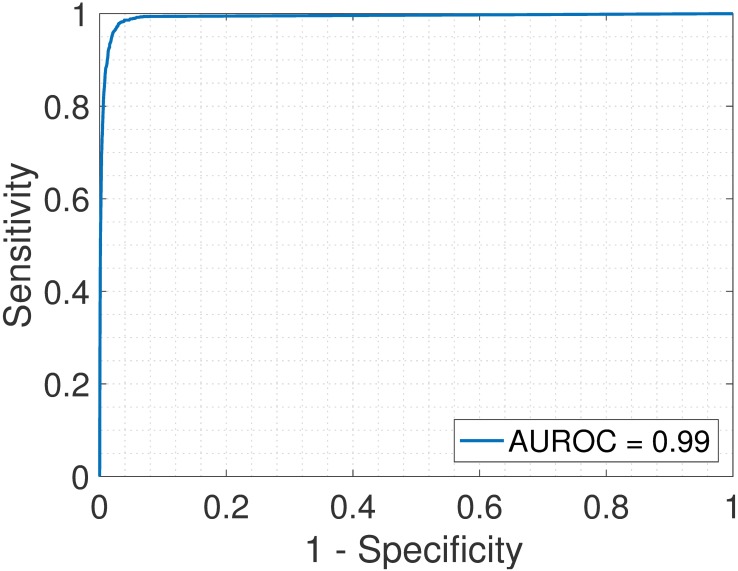
Brain > Heart. ROC plot obtained from the proposed SMC method for brain vs. heart cell types, brain upregulated (affymetrix dataset).

**Fig 6:**
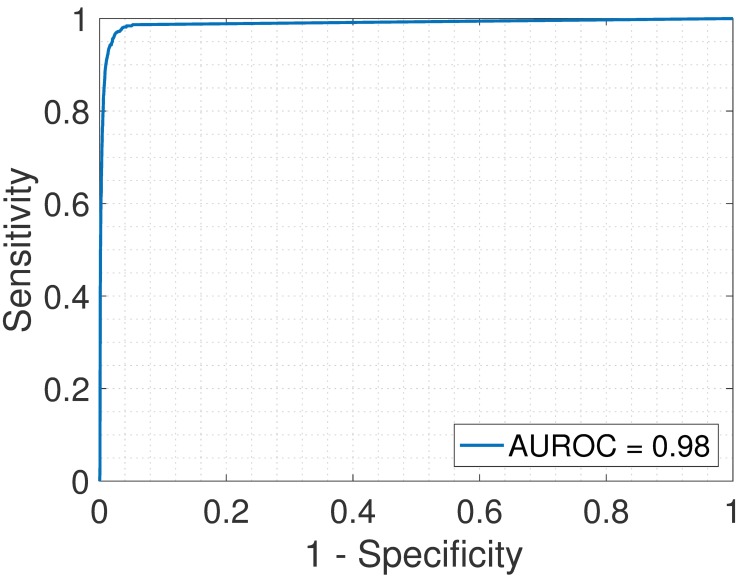
Heart > Brain. ROC plot obtained from the proposed SMC method for brain vs. heart cell types, heart upregulated (affymetrix dataset).

**Fig 7:**
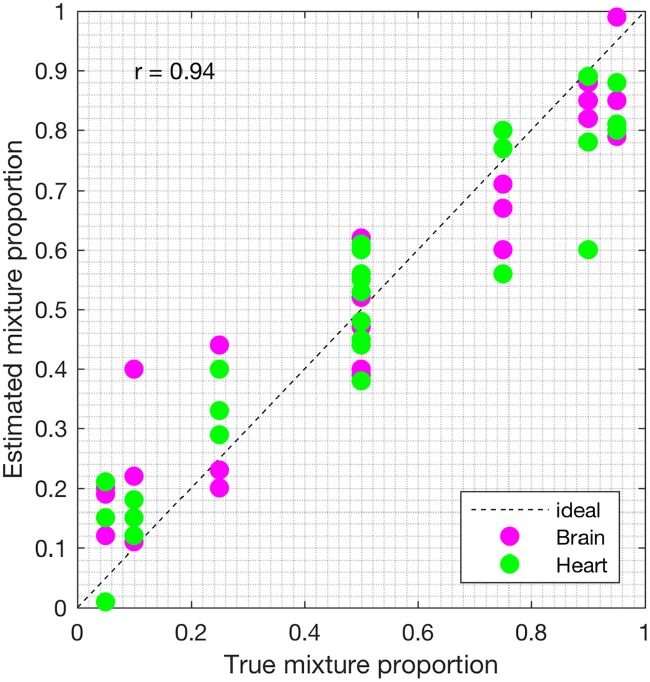
Plot of proportions. Plot of the true proportions vs. estimated proportions obtained from the MCMC method (affymetrix dataset).

**Fig 8:**
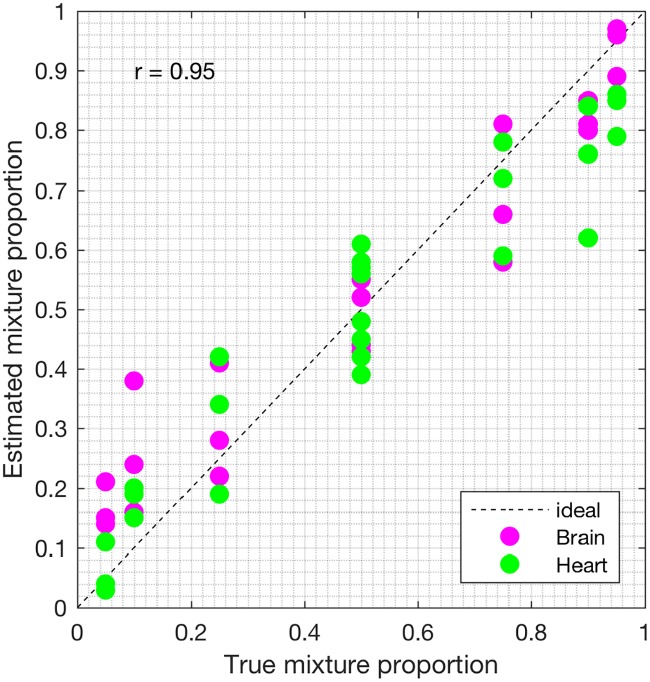
Plot of proportions. Plot of the true proportions vs. estimated proportions obtained from the PNMF method (affymetrix dataset).

First, we analyzed the heterogeneous gene expression matrix **Y** with the SMC method and the plot of the estimated proportions, matrix $\hat{M}$ versus the true proportions, matrix **M** is shown in Fig 4 with the Pearson correlation coefficient, *r* = 0.99. In Table 3, we record the correlation between the true and the estimated cell-type specific expression profiles for all the cell types. Further, we test the power of the SMC method to detect truly differentially expressed and non-differentially expressed genes between cell types. Figs 5 and 6 show the ROCs generated with the SMC method and the AUROC for each plot is recorded in Table 3. Moreover, we analyzed the same dataset with the MCMC method and the PNMF algorithms and the results are presented in Figs 7 and 8, and in Table 3. The results obtained and presented in Table 3 show that the proposed SMC method accurately estimates cell type proportions, cell-type specific expressions and in fact, more specific in identifying the differentially expressed genes when compared to the other two methods.

**Table 3:** Pearson correlation coefficient (*r*) and AUROC for the affymetrix dataset (AUROC in columns 3 and 4).

	*r*_*M*_	*r*_*B*_	*r*_*H*_	Brain > Heart	Heart > Brain
SMC	0.99	0.98	0.98	0.99	0.98
MCMC	0.93	0.92	0.94	0.91	0.92
PNMF	0.95	0.95	0.95	0.96	0.94

*r*_*M*_, *r*_*B*_ and *r*_*H*_ denote the Pearson correlation coefficients between the true and the estimated: (i) cell types proportions, (ii) the brain cell expression profiles, and (iii) the heart cell expression profiles, respectively. In columns 5 and 6, Brain > Heart, for example, implies that brain is upregulated as compared to heart.

### GEO series GSE19830 dataset: 3 cell types

In the mixture experiment by [7], tissue samples from the liver, brain and lung of a single rat were analyzed using Affymetrix expression arrays. Biospecimens from the three different tissues were mixed in different proportions (mixture proportion of each sample is shown in Table B in S1 Supplementary Material). The data consists of 11 different mixtures, each mixture with 3 technical replicates. In addition, there are 9 samples for the pure tissues (S1—S9), 3 technical replicates for each pure tissue type. We downloaded the dataset from the NCBI GEO website and performed data preprocessing with the RMA.

**Fig 9:**
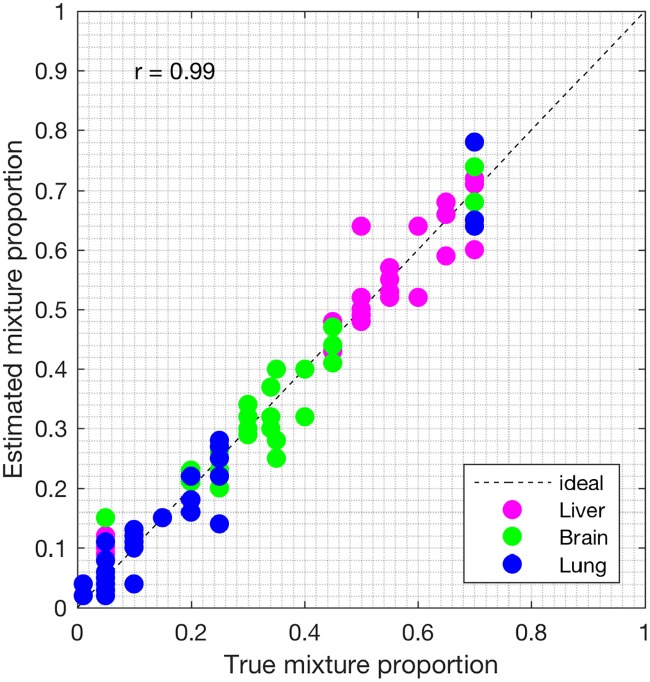
Plot of proportions. Plot of the true proportions vs. estimated proportions obtained from the proposed SMC method (GSE19830 dataset).

**Fig 10:**
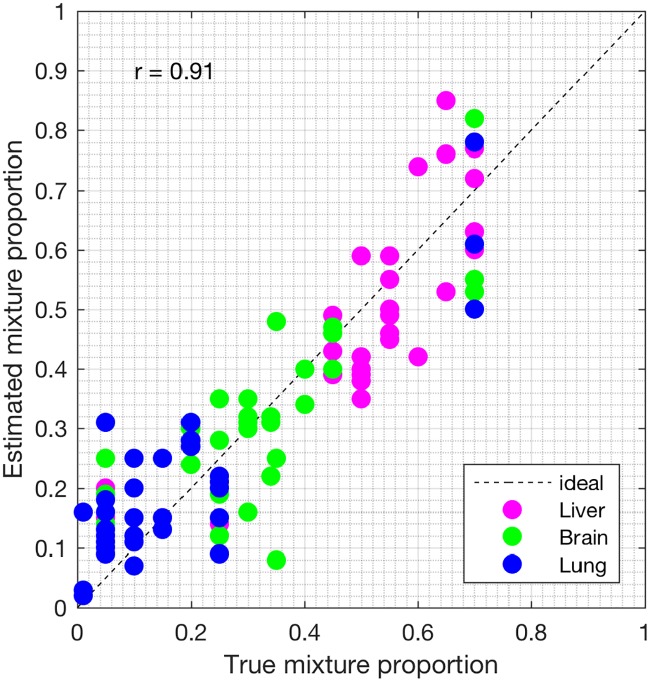
Plot of proportions. Plot of the true proportions vs. estimated proportions obtained from the MCMC method (GSE19830 dataset).

**Fig 11:**
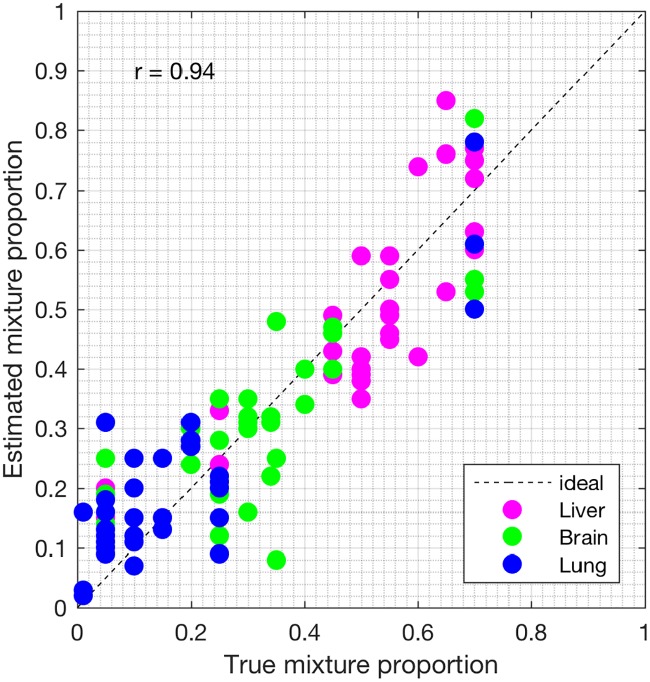
Plot of proportions. Plot of the true proportions vs. estimated proportions obtained from the PNMF method (GSE19830 dataset).

**Fig 12:**
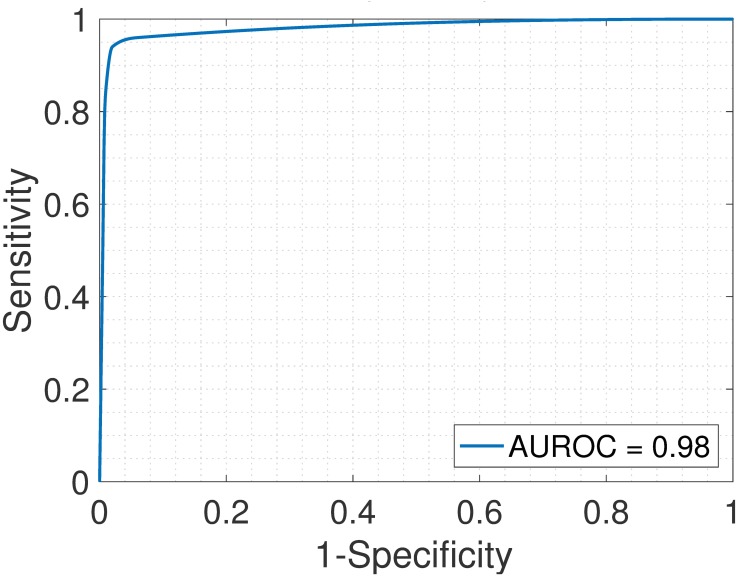
Liver > Brain. ROC plot obtained from the proposed SMC method for liver vs. brain cell types, liver upregulated (GSE19830 dataset).

**Fig 13:**
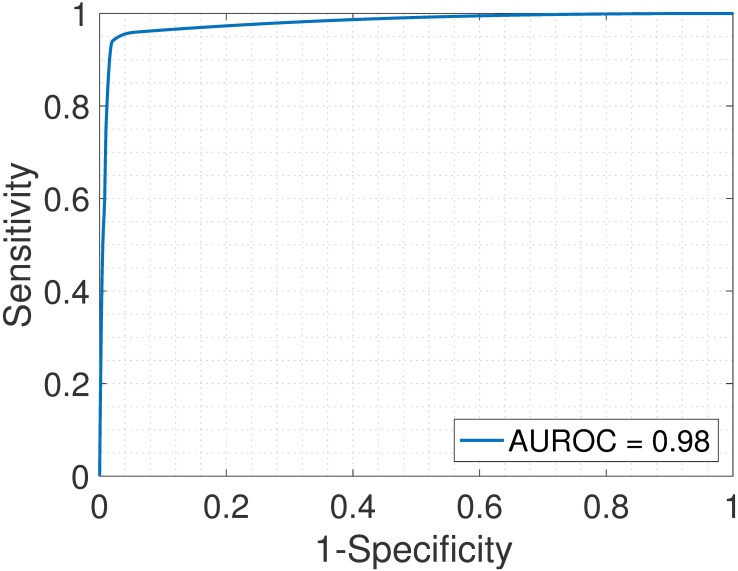
Liver > Lung. ROC plot obtained from the proposed SMC method for liver vs. lung cell types, liver upregulated (GSE19830 dataset).

**Fig 14:**
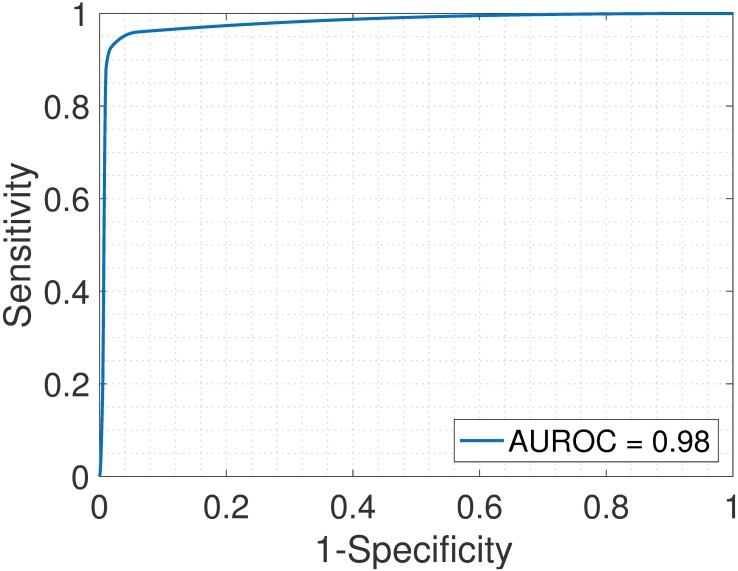
Brain > Lung. ROC plot obtained from the proposed SMC method for brain vs. lung cell types, brain upregulated (GSE19830 dataset).

We analyzed the heterogeneous gene expression matrix with the SMC method and the plot of the estimated proportions, matrix $\hat{M}$ versus the true proportions, matrix **M** is shown in Fig 9 with the Pearson correlation coefficient, *r* = 0.99 (similar results are obtained for the MCMC and the PNMF methods in Figs 10 and 11, respectively). In addition, we record the correlation between the true and the estimated cell-type specific expression profiles in Table 4. Next, on this dataset, we test the power of the SMC method to detect truly differentially expressed and non-differentially expressed genes between cell types. Figs 12, 13 and 14 (and Fig A in S1 Supplementary Material) show the ROCs generated with the SMC method and the AUROC for each plot is recorded in Table 5. Moreover, we analyzed same dataset with the MCMC method and the PNMF algorithm and the results for the correlations and AUROC are presented in Tables 4 and 5, respectively. The results obtained show that the proposed SMC method accurately estimates cell type proportions, cell-type specific expressions and in fact, more specific in identifying the differentially expressed and non-differentially expressed genes when compared to the two other methods.

**Table 4:** Pearson correlation coefficient (*r*) for the GSE19830 dataset.

	*r*_*M*_	*r*_*Li*_	*r*_*Br*_	*r*_*Lu*_
SMC	0.99	0.98	0.95	0.98
MCMC	0.91	0.90	0.91	0.89
PNMF	0.94	0.93	0.93	0.94

*r*_*M*_, *r*_*Li*_, *r*_*Br*_ and *r*_*Lu*_ denote the Pearson correlation coefficients between the true and the estimated: (i) cell types proportions, (ii) the liver cell expression profiles, (iii) the brain cell expression profiles, and (iv) the lung cell expression profiles, respectively.

**Table 5:** AUROC for the GSE19830 dataset.

	Liver > Brain	Liver > Lung	Brain > Lung	Liver < Brain	Liver < Lung	Brain < Lung
SMC	0.98	0.98	0.98	0.98	0.97	0.98
MCMC	0.90	0.89	0.91	0.88	0.90	0.91
PNMF	0.93	0.94	0.94	0.93	0.95	0.95

For example, Liver > Brain implies that liver is upregulated as compared to brain.

### GEO series GSE11058 dataset: 4 cell types

In the real mixtures with 2 and 3 cell types, expression differences between different cell types are relatively higher compared to the expression differences between cell types within a tissue sample. Hence, we tested the proposed algorithm on real tissue samples that are composed of cell types with gene expression profiles that are more similar to each other. Specifically, we analyzed a publicly available dataset from the GEO series GSE11058, downloaded from the NCBI GEO [54] and data preprocessing was done by RMA. Each heterogeneous sample in the data comprises of 4 different cell lines of immune origin, namely: Jurkat (J), IM-9 (I), Raji (R) and THP-1 (T). In total, there are 24 samples in the dataset, that is, triplicates of each pure cell type and four different mixtures for which the relative proportions of each cell type are known, as shown in Table C in S1 Supplementary Material where samples are designated S1,…,S24 for sample 1,…,sample 24, respectively. The first 12 samples, samples from pure cell types constitute the matrix $\tilde{Y}$, which is used for approximating the “ground-truths” for the cell-type expression profiles (matrix **X**), marker probesets and the list of truly differentially expressed and non-differentially expressed genes.

**Fig 15:**
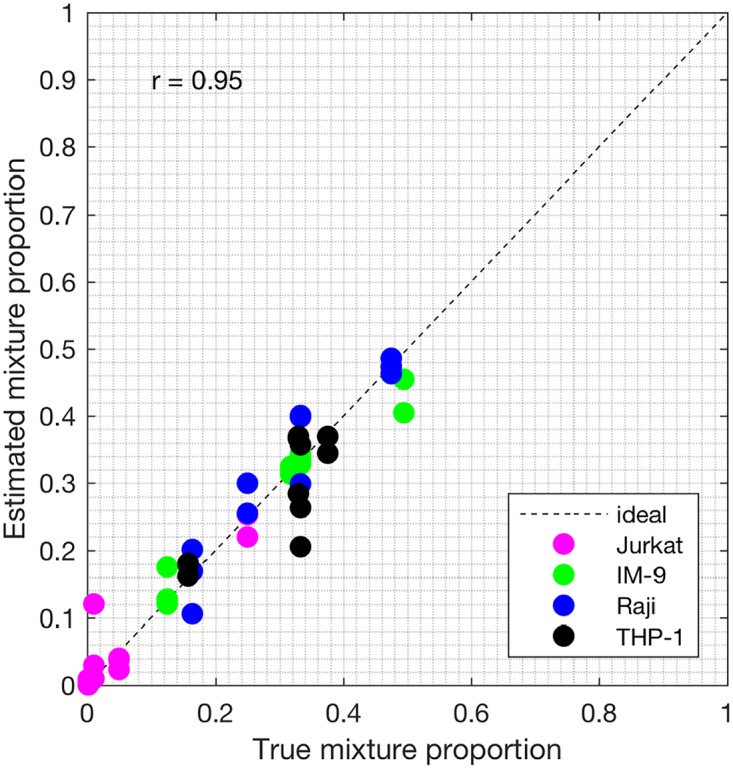
Plot of proportions. Plot of the true proportions vs. estimated proportions obtained from the proposed SMC method (GSE11058 dataset).

**Fig 16:**
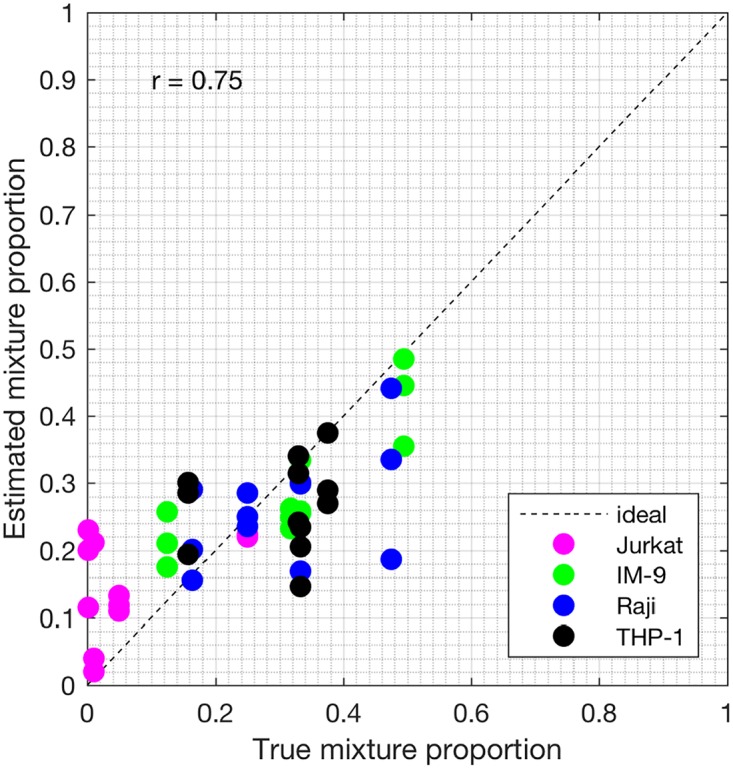
Plot of proportions. Plot of the true proportions vs. estimated proportions obtained from the proposed MCMC method (GSE11058 dataset).

**Fig 17:**
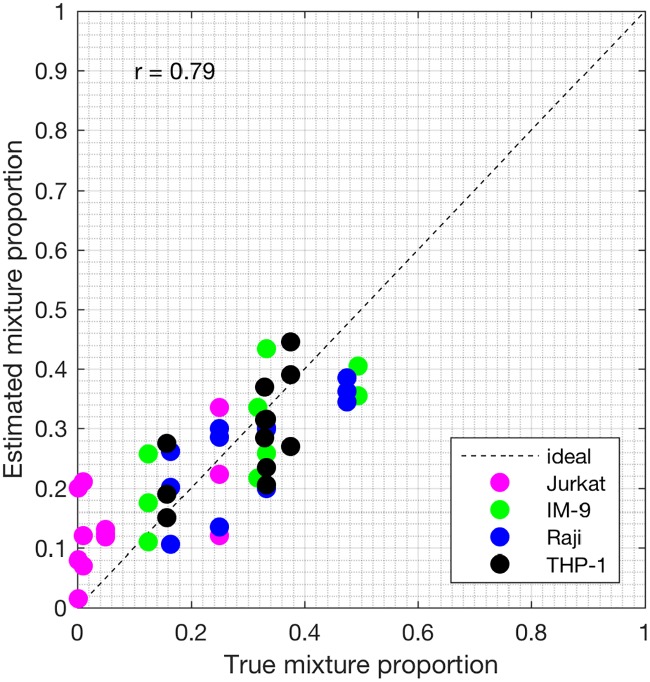
Plot of proportions. Plot of the true proportions vs. estimated proportions obtained from the proposed PNMF method (GSE11058 dataset).

**Fig 18:**
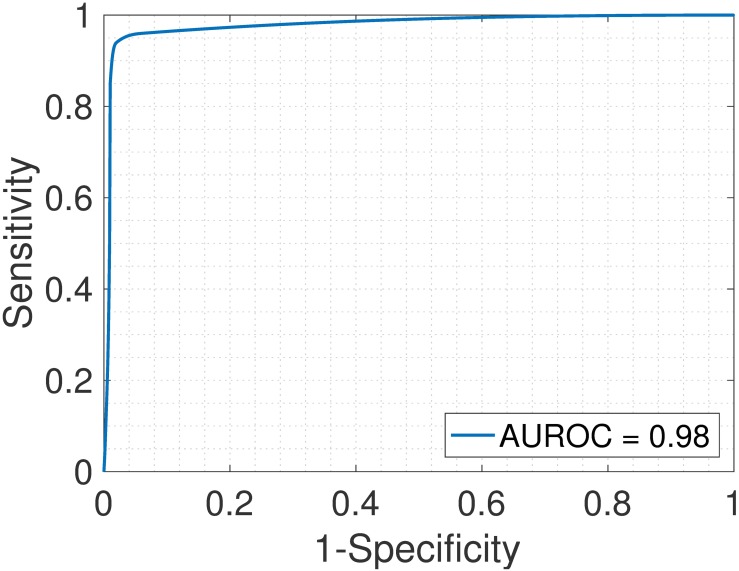
Jurkat > IM-9. ROC plot obtained from the proposed SMC method for Jurkat vs. IM-9 cell types, Jurkat upregulated (GSE11058 dataset).

**Fig 19:**
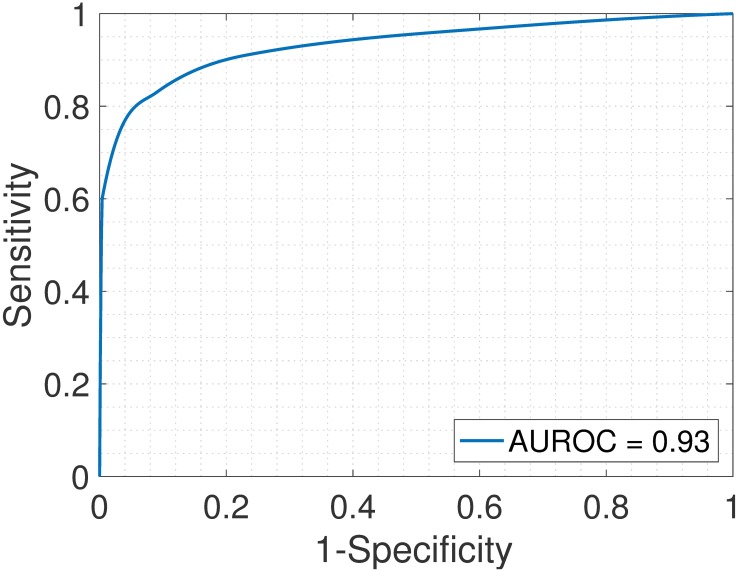
Jurkat > Raji. ROC plot obtained from the proposed SMC method for Jurkat vs. Raji cell types, Jurkat upregulated (GSE11058 dataset).

**Fig 20:**
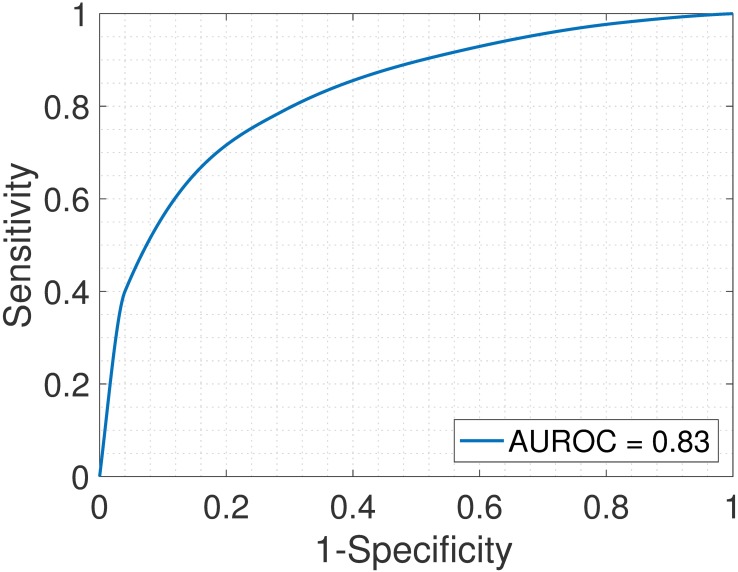
Jurkat > THP-1. ROC plot obtained from the proposed SMC method for Jurkat vs. THP-1 cell types, Jurkat upregulated (GSE11058 dataset).

Samples S13—S24 constitute the heterogeneous gene expression matrix **Y** that we analyzed with the proposed SMC method, the MCMC method and the PNMF method. Figs 15, 16 and 17 and Table 6 show the correlation values obtained between the estimated cellular proportions and the true proportions, and then the estimated cell-type specific expression profiles and the true expression profiles. In addition, AUROC for all methods is shown in Table 7 and the ROC plots obtained for the proposed SMC method are shown in Figs 18, 19 and 20 and in Figs B and C in S1 Supplementary Material. Again, the SMC method outperformed the MCMC method and the PNMF method in terms of the accuracy of the cellular proportions estimates and the cell-type specific expression estimates, and finally, in identifying differentially and non-differentially expressed genes.

**Table 6:** Pearson correlation coefficient (*r*) for the GSE19830 dataset.

	*r*_*M*_	*r*_*J*_	*r*_*I*_	*r*_*R*_	*r*_*T*_
SMC	0.99	0.97	0.98	0.98	0.96
MCMC	0.91	0.90	0.90	0.91	0.92
PNMF	0.94	0.93	0.95	0.93	0.94

*r*_*M*_, *r*_*J*_, *r*_*I*_, *r*_*R*_, and *r*_*T*_ denote the Pearson correlation coefficients between the true and the estimated: (i) cell types proportions, (ii) the Jurkat cell expression profiles, (iii) the IM-9 cell expression profiles, (iv) the Raji cell expressions profiles, and (iv) the THP-1 cell expression profiles, respectively.

**Table 7:** AUROC for the GSE19830 dataset.

	J>I	J>R	J>T	I>R	I>T	R>T	J<I	J<R	J<T	I<R	I<T	R<T
SMC	0.98	0.93	0.83	0.93	0.89	0.93	0.92	0.90	0.87	0.95	0.96	0.96
MCMC	0.90	0.89	0.91	0.88	0.90	0.91	0.92	0.91	0.91	0.89	0.92	0.91
PNMF	0.93	0.94	0.94	0.93	0.95	0.92	0.94	0.94	0.94	0.93	0.95	0.95

J = Jurkat; I = IM-9; R = Raji; T = THP-1. For example, J > I implies that Jurkat is upregulated as compared to IM-9.

## Discussion

In this paper, we modeled the heterogeneous gene expression data using a Bayesian framework. Specifically, we modeled the expression of a gene in each sample as the sum of expressions of that gene in all the constituting cell types in the sample, weighted by the proportions of all cell types in the sample plus an additive Gaussian noise.

We proposed an efficient SMC algorithm, a novel Bayesian approach that is based on sampling technology suited for approximating the posterior distributions of complex model parameters. In this paper, we obtained the estimates of the cellular proportions (matrix **M**) and the cell-type specific expression profiles (matrix **X**) from the heterogeneous gene expression data. Further, the estimated expression profiles are used to identify genes that are differentially expressed which is one of the major reasons for carrying out gene expression deconvolution analysis. In addition to the identification of the differentially expressed genes, performing the complete gene expression deconvolution is an attractive method that provides an alternative to the very expensive and time consuming manual approaches like LCM and flow cytometry for separating cells which often lead to an altered cell-type specific gene expression profiles. Unlike some previously proposed methods for gene expression data deconvolution, our method does not rely on any prior knowledge of the cell type proportions or the cell-type specific gene expression profiles.

In testing the performance of the proposed SMC method, we evaluated the method on simulated datasets and publicly available real datasets. From the results obtained in all the experiments, the proposed SMC method demonstrated a superior performance in terms of accuracy of the estimated model parameters and also in identifying differentially expressed genes as shown in the Results Section and in the S1 Supplementary Material, when compared to the two other methods.

Moreover, in mapping the estimated cell-type specific profiles (matrix $\hat{X}$) to the true cell types, we defined a set of marker probesets which were defined from the gene expression data from pure samples, matrix $\tilde{Y}$. Although in the real settings, we have no access to these pure samples, a small number of cell-type specific markers are often available, for instance, [55] identified a set of markers for different immune subsets.

Finally, it was shown that PNMF and the MCMC methods are faster than the SMC method in terms of computational speed. However, when there is an option of parallelization of computational resources, the SMC method can be considerably improved in terms of the computational time.

## Supporting information

S1 Supplementary Material Supplementary Material for “A Sequential Monte Carlo Approach to Gene Expression Deconvolution”. (PDF)
