# Supplementary material for: A sequential Monte Carlo approach to gene expression deconvolution
Source: PLoS One. 2017 Oct 19;12(10):e0186167. doi: 10.1371/journal.pone.0186167 (PMC5648148; doi:10.1371/journal.pone.0186167)
Supplement: S1 Supplementary Material — (PDF) [file pone.0186167.s001.pdf]

Supplementary Material for " A Sequential Monte Carlo Approach to Gene Expression Deconvolution"

Oyetunji E. Ogundijo and Xiaodong Wang

Probabilistic Model and Sequence of Target Distributions

Here, we give the data generating probabilistic model and subsequently, show the detailed derivations of the sequence of target distributions for all the unknown model parameters for gene expression decomposition.

Data Likelihood

From (4), the joint data likelihood can be written as:

$$p(\mathbf{Y}|\boldsymbol{\theta}) = \prod_{i=1}^{I}\prod_{j=1}^{J} p\left(y_{ij}\middle|\sum_{k=1}^{K} x_{ik}m_{kj}, \lambda\right),$$

where $\boldsymbol{\theta} = \{\lambda, x_{ik}, m_{kj} : i = 1, ..., I, j = 1, ..., J, k = 1, ..., K\}$ are the unknown model parameters.

Prior Densities

Prior densities for all the model parameters are given as follows:

Cell type proportions

$$m_{kj} \sim \mathcal{N}(\mu_{kj}, \nu_{kj}^{-1}),$$

μ_{kj} is chosen as 0 and ν_{kj} is chosen as 1/0.01 ,$k = 1, ..., K,\ j = 1, ..., J$.

Cell-type specific expression

$$x_{ik} \sim \mathcal{N}(\mu_{ik}, \nu_{ik}^{-1}),$$

μ_{ik} is chosen as $\mathcal{U}(0, 100)$ and ν_{ik} is chosen as 1/1000, $i = 1, ..., I,\ k = 1, ..., K$.

Precision

$$\lambda \sim \text{Gamma}(\alpha, \beta),$$

where $\alpha = 2$ and $\beta = 10000$.

Moreover, we adopted a linear cooling schedule where $\epsilon_1 = 0$, $\epsilon_T = 1$, $\Delta\epsilon = \epsilon_t - \epsilon_{t-1} = 10^{-4}$. Also, $N = 40$ (number of samples) was used for all the experiments.

Derivation of the Sequence of Target Distributions for Model Parameters

Define:

$$\mathcal{Y}_{ijk} = \sum_{k' \neq k} x_{ik'} m_{k'j},$$
$$i = 1, ..., I, j = 1, ..., J, k = 1, ..., K.$$

Next, we show the sequence of target distributions for the unknown model parameters.

Sequence of Target Distributions for cell type proportions

$$
\begin{aligned}
\pi_t(m_{kj}|\cdot) &\propto p(m_{kj}|\mu_{kj},\nu_{kj})\Bigg[\prod_{i=1}^{I} p\Big(y_{ij}|\sum_{k'=1}^{K} x_{ik'}m_{k'j},\lambda\Big)\Bigg]^{\epsilon_t} \\
&\propto \exp\Bigg\{-\frac{\nu_{kj}}{2}(m_{kj}-\mu_{kj})^2-\frac{\epsilon_t\lambda}{2}\sum_{i=1}^{I}\Bigg(y_{ij}-\sum_{k'=1}^{K} x_{ik'}m_{k'j}\Bigg)^2\Bigg\} \\
&\propto \exp\Bigg\{-\frac{\nu_{kj}}{2}(m_{kj}-\mu_{kj})^2-\frac{\epsilon_t\lambda}{2}\sum_{i=1}^{I}\Big(m_{kj}^2x_{ik}^2-2m_{kj}y_{ij}x_{ik}+2m_{kj}\mathcal{Y}_{ijk}x_{ik}\Big)\Bigg\} \\
&\propto \exp\Bigg\{-\frac{1}{2}\Bigg[m_{kj}^2\Bigg(\nu_{kj}+\epsilon_t\lambda\sum_{i=1}^{I}x_{ik}^2\Bigg)-2m_{kj}\Bigg(\mu_{kj}\nu_{kj}+\epsilon_t\lambda\sum_{i=1}^{I}y_{ij}x_{ik}-\epsilon_t\lambda\sum_{i=1}^{I}\mathcal{Y}_{ijk}x_{ik}\Bigg)\Bigg]\Bigg\} \\
&= \exp\Bigg\{-\frac{1}{2}\Bigg[m_{kj}^2U_{kj}^t-2m_{kj}V_{kj}^t\Bigg]\Bigg\} \\
&= \exp\Bigg\{-\frac{U_{kj}^t}{2}\Bigg[m_{kj}^2-2m_{kj}\frac{V_{kj}^t}{U_{kj}^t}\Bigg]\Bigg\} \\
&\propto \exp\Bigg\{-\frac{U_{kj}^t}{2}\Bigg[m_{kj}^2-2m_{kj}\frac{V_{kj}^t}{U_{kj}^t}+\Bigg(\frac{V_{kj}^t}{U_{kj}^t}\Bigg)^2\Bigg]\Bigg\} \\
&= \exp\Bigg\{-\frac{U_{kj}^t}{2}\Bigg(m_{kj}-\frac{V_{kj}^t}{U_{kj}^t}\Bigg)^2\Bigg\}.
\end{aligned}
$$

Thus,

$$
\pi_t(m_{kj}|\cdot) = \mathcal{N}\Bigg(\frac{V_{kj}^t}{U_{kj}^t},\frac{1}{U_{kj}^t}\Bigg),
$$

$$
\text{where } U_{kj}^t=\nu_{kj}+\epsilon_t\lambda\sum_{i=1}^{I}x_{ik}^2 \text{ and } V_{kj}^t=\mu_{kj}\nu_{kj}+\epsilon_t\lambda\Bigg(\sum_{i=1}^{I}y_{ij}x_{ik}-\sum_{i=1}^{I}\mathcal{Y}_{ijk}x_{ik}\Bigg),
$$

$$
k=1,...,K, j=1,...,J, t=1,...,T.
$$

Sequence of Target Distributions for the cell-type specific expressions

$$
\begin{aligned}
\pi_t(x_{ik}|\cdot) &\propto p(x_{ik}|\mu_{ik},\nu_{ik})\left[\prod_{j=1}^{J} p\bigg(y_{ij}|\sum_{k'=1}^{K} x_{ik'}m_{k'j},\lambda\bigg)\right]^{\epsilon_t} \\
&\propto \exp\left\{-\frac{\nu_{ik}}{2}(x_{ik}-\mu_{ik})^2-\epsilon_t\lambda\sum_{j=1}^{J}\left(y_{ij}-\sum_{k'=1}^{K} x_{ik'}m_{k'j}\right)^2\right\} \\
&\propto \exp\left\{-\frac{\nu_{ik}}{2}(x_{ik}-\mu_{ik})^2-\frac{\epsilon_t\lambda}{2}\sum_{j=1}^{J}\left(x_{ij}^2m_{kj}^2-2x_{ik}y_{ij}m_{kj}+2x_{ik}\mathcal{Y}_{ijk}m_{kj}\right)\right\} \\
&\propto \exp\left\{-\frac{1}{2}\left[x_{ik}^2\bigg(\nu_{ik}+\epsilon_t\lambda\sum_{j=1}^{J} m_{kj}^2\bigg)-2x_{ik}\bigg(\mu_{ik}\nu_{ik}+\epsilon_t\lambda\sum_{j=1}^{J} y_{ij}m_{kj}-\epsilon_t\lambda\sum_{j=1}^{J}\mathcal{Y}_{ijk}m_{kj}\bigg)\right]\right\} \\
&= \exp\left\{-\frac{1}{2}\left[x_{ik}^2A_{ik}^t-2x_{ik}B_{ik}^t\right]\right\} \\
&= \exp\left\{-\frac{A_{ik}^t}{2}\left[x_{ik}^2-2x_{ik}\frac{B_{ik}^t}{A_{ik}^t}\right]\right\} \\
&\propto \exp\left\{-\frac{A_{ik}^t}{2}\left[x_{ik}^2-2x_{ik}\frac{B_{ik}^t}{A_{ik}^t}+\left(\frac{B_{ik}^t}{A_{ik}^t}\right)^2\right]\right\} \\
&= \exp\left\{-\frac{A_{ik}^t}{2}\left[x_{ik}-\frac{B_{ik}^t}{A_{ik}^t}\right]^2\right\}.
\end{aligned}
$$

Thus,

$$
\pi_t(x_{ik}|\cdot) = \mathcal{N}\left(\frac{B_{ik}^t}{A_{ik}^t},\frac{1}{A_{ik}^t}\right),
$$

$$
\text{where } A_{ik}^t=\nu_{ik}+\epsilon_t\lambda\sum_{j=1}^{J} m_{kj}^2 \text{ and } B_{ik}^t=\mu_{ik}\nu_{ik}+\epsilon_t\lambda\left(\sum_{j=1}^{J} y_{ij}m_{kj}-\sum_{j=1}^{J}\mathcal{Y}_{ijk}m_{kj}\right)),
$$

$$
i=1,...,I,k=1,...,K,t=1,...,T.
$$

Sequence of Target Distributions for the precision

$$\pi_t(\lambda|\cdot) \propto p(\lambda|\alpha,\beta)\Bigg[\prod_{i=1}^{I}\prod_{j=1}^{J} p\Big(y_{ij}|\sum_{k=1}^{K}x_{ik}m_{kj},\lambda\Big)\Bigg]^{\epsilon_t}$$

$$\propto \lambda^{\alpha-1}\exp\{-\lambda\beta\}\Bigg[\lambda^{\frac{IJ}{2}}\exp\Bigg\{-\frac{\lambda}{2}\sum_{i=1}^{I}\sum_{j=1}^{J}\Big(y_{ij}-\sum_{k=1}^{K}x_{ik}m_{kj}\Big)^2\Bigg\}\Bigg]^{\epsilon_t}$$

$$= \lambda^{\alpha-1}\exp\{-\lambda\beta\}\lambda^{\frac{\epsilon_t IJ}{2}}\exp\Bigg\{-\frac{\lambda}{2}\sum_{i=1}^{I}\sum_{j=1}^{J}\Big(y_{ij}-\sum_{k=1}^{K}x_{ik}m_{kj}\Big)^2\Bigg\}$$

$$= \lambda^{(\alpha+\frac{\epsilon_t IJ}{2})-1}\exp\Bigg\{-\lambda\Bigg(\beta+\frac{\epsilon_t}{2}\sum_{i=1}^{I}\sum_{j=1}^{J}\Big(y_{ij}-\sum_{k=1}^{K}x_{ik}m_{kj}\Big)^2\Bigg)\Bigg\}.$$

Thus,

$$\pi_t(\lambda|\cdot) = \text{Gamma}\big(\tilde{\alpha},\tilde{\beta}\big),$$

$$\text{where }\ \tilde{\alpha} = \alpha + \frac{\epsilon_t IJ}{2}\ \text{ and }\ \tilde{\beta} = \beta + \frac{\epsilon_t}{2}\sum_{i=1}^{I}\sum_{j=1}^{J}\Big(y_{ij}-\sum_{k=1}^{K}x_{ik}m_{kj}\Big)^2),$$

$$t = 1,...,T.$$

Importance Weights for SMC Samplers

From (10), the unnormalized weights at time t is given as:

$$\tilde{w}_t^n \propto \tilde{w}_{t-1}^n W_t(\boldsymbol{\theta}_{t-1}^n,\boldsymbol{\theta}_t^n)$$

with $\{\tilde{w}_{t-1}^n\}_{n=1}^N$ the unnormalized weights at time $t-1$ and $\{W_t(\boldsymbol{\theta}_{t-1}^n,\boldsymbol{\theta}_t^n)\}_{n=1}^N$, the unnormalized incremental weights, calculated as:

$$W_t(\boldsymbol{\theta}_{t-1}^n,\boldsymbol{\theta}_t^n) = p(\mathbf{Y}|\boldsymbol{\theta}_{t-1}^n)^{(\epsilon_t-\epsilon_{t-1})},\ \ n=1,...,N$$

Table A: Known cell type proportions for each sample in Affymetrix dataset

	S1-S3	S4-S6	S7-S9	S10-S12	S13-S21	S22-S24	S25-S27	S28-S30	S31-S33
Brain	0.00	0.05	0.10	0.25	0.50	0.75	0.90	0.95	1.00
Heart	1.00	0.95	0.90	0.75	0.50	0.25	0.10	0.05	0.00

Table B: Known cell type proportions for each sample in the GSE19830 dataset

	Samples	Tissue type	Liver	Brain	Lung
1	S1 - S3	Pure	1.00	0.00	0.00
2	S4 - S6	Pure	0.00	1.00	0.00
3	S7 - S9	Pure	0.00	0.00	1.00
4	S10 - S12	Mixed	0.05	0.25	0.70
5	S13 - S15	Mixed	0.70	0.05	0.25
6	S16 - S18	Mixed	0.25	0.70	0.05
7	S19 - S21	Mixed	0.70	0.25	0.05
8	S22 - S24	Mixed	0.45	0.45	0.10
9	S25 - S27	Mixed	0.55	0.20	0.25
10	S28 - S30	Mixed	0.50	0.30	0.20
11	S31 - S33	Mixed	0.55	0.30	0.15
12	S34 - S36	Mixed	0.50	0.40	0.10
13	S37 - S39	Mixed	0.60	0.35	0.05
14	S40 - S42	Mixed	0.65	0.34	0.01

where

$$\begin{aligned}
p(\mathbf{Y}|\boldsymbol{\theta})^{\epsilon_t-\epsilon_{t-1}} &= \left[\prod_{i=1}^{I}\prod_{j=1}^{J} p\left(y_{ij}\middle|\sum_{k=1}^{K} x_{ik}m_{kj}, \lambda^{-1}\right)\right]^{\epsilon_t-\epsilon_{t-1}} \\
&= \left[\left(\sqrt{\frac{\lambda}{2\pi}}\right)^{IJ} \exp\left\{\frac{\lambda}{2}\sum_{i=1}^{I}\sum_{j=1}^{J}\left(y_{ij}-\sum_{k=1}^{K} x_{ik}m_{kj}\right)^2\right\}\right]^{\epsilon_t-\epsilon_{t-1}} \\
&\propto \lambda^{\frac{IJ(\epsilon_t-\epsilon_{t-1})}{2}} \exp\left\{-\frac{(\epsilon_t-\epsilon_{t-1})\lambda}{2}\sum_{i=1}^{I}\sum_{j=1}^{J}\left(y_{ij}-\sum_{k=1}^{K} x_{ik}m_{kj}\right)^2\right\}, \\
&\qquad t = 1, ..., T.
\end{aligned}$$

Table C: Known cell type proportions for each sample in the GSE11058 dataset

	S1-S3	S4-S6	S7-S9	S10-S12	S13-S15	S16-S18	S19-S21	S22-S24
Jurkat	1.000	0.000	0.000	0.000	0.250	0.500	0.010	0.002
IM-9	0.000	1.000	0.000	0.000	0.125	0.317	0.497	0.333
Raji	0.000	0.000	1.000	0.000	0.250	0.475	0.165	0.333
THP-1	0.000	0.000	0.000	1.000	0.375	0.158	0.330	0.333

Marker Probesets and Cell Types Mapping

Marker Probesets

We obtained the list of marker probsets as follows. Given the matrices $\tilde{\mathbf{Y}}$ and $\mathbf{X}$, for $i = 1, ..., I$, define $\tilde{\mathbf{y}}_i = [\tilde{\mathbf{y}}_i^1, \tilde{\mathbf{y}}_i^2, ..., \tilde{\mathbf{y}}_i^K]$ and $[\mathbf{r}_i^{1:2}, \mathbf{s}_i^{1:2}] = \text{sort}^d(\mathbf{x}_i)$, where $\tilde{\mathbf{y}}_i$ is the ith row of matrix $\tilde{\mathbf{Y}}$, $\mathbf{x}_i$ is the ith row of matrix $\mathbf{X}$, $\mathbf{r}_i^{1:2}$ is the first 2 values after the entries of $\mathbf{x}_i$ have been sorted in a descending order and $\mathbf{s}_i^{1:2}$ are the indices of the values in $\mathbf{r}_i^{1:2}$ before sorting. Then, we check the following: (i) $\log_2(\max(\mathbf{r}_i^{1:2})/\min(\mathbf{r}_i^{1:2}))$ is greater than 1.5, and (ii) p-value (using a two-sided t-test with equal variance) computed on $\log_2$ transformation of row vectors $\tilde{\mathbf{y}}_i^c$ and $\tilde{\mathbf{y}}_i^d$, c and d being the entries of $\mathbf{s}_i^{1:2}$, is less than 0.05 (significant level). If both conditions are true, then we include probeset i in the marker list for cell type c. In addition, we defined marker probesets for the simulated datasets similar to the method proposed by ([1]). Here, each row of the pure cell-type gene expression profiles, matrix $\mathbf{X}$ is divided by its sum, so that after the normalization, every row of matrix $\mathbf{X}$ sums to 1. Thus, a value close to 1 implies that such probeset is highly expressed in that particular cell type and it is included in the marker list for the cell type. In summary, in all datasets, each defined marker probeset is associated with a specific cell type (true map).

Cell types mapping:

To do the mapping of the estimated profiles, that is, each column of matrix $\hat{\mathbf{X}}$ to the correct cell types, i.e., columns of matrix $\mathbf{X}$, we follow the mapping strategy proposed in ([2]). First, we build a predicted map where each defined marker is associated with the column of matrix $\hat{\mathbf{X}}$ that expresses it the most. Next, a contingency table is constructed using the true map of the markers to cell types and the predicted map. Specifically, each entry in

the contingency table is the number of markers that are consistent between a given column of the estimated matrix $\hat{\mathbf{X}}$ and a given column of the true matrix $\mathbf{X}$. The columns of the contingency table are then scaled to sum to 1 so as to obtain the percentages of markers from each column of $\mathbf{X}$ that are consistent with each column of matrix $\hat{\mathbf{X}}$. The column of $\hat{\mathbf{X}}$ and the column of $\mathbf{X}$ that achieve the maximum percentage of consistent markers are then mapped together and removed from the table. The mapping is repeated until all the columns of $\hat{\mathbf{X}}$ and the columns of $\mathbf{X}$ have been completely mapped.

References

[1] Venet,D. et al. (2001) Separation of samples into their constituents using gene expression data. *Bioinformatics*, **17**, S279–S287.

[2] Gaujoux,R., Seoighe,C. (2012) Semi-supervised Nonnegative Matrix Factorization for gene expression deconvolution: a case study. *Infection, Genetics and Evolution*, **12**, 913–921.

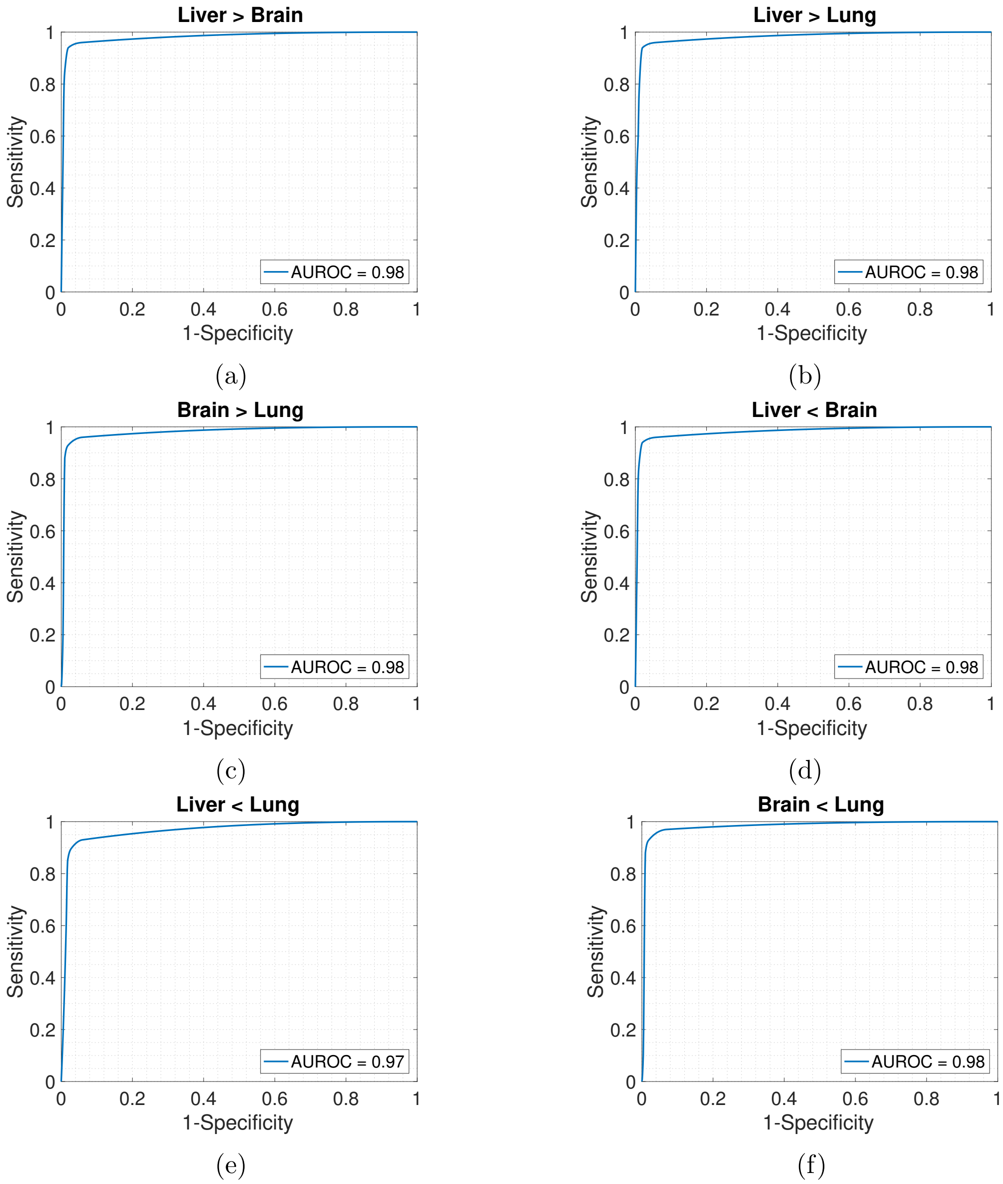

Fig A: Performance evaluation of the SMC method with the GSE19830 dataset. ROC plot is shown for all the cell types. For example, Liver > Brain implies that Liver is upregulated as compared to Brain.

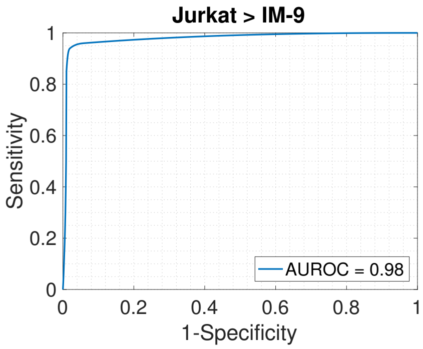

(a)

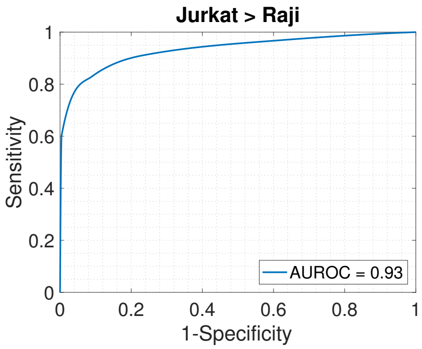

(b)

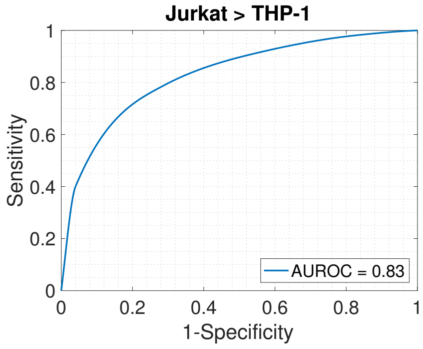

(c)

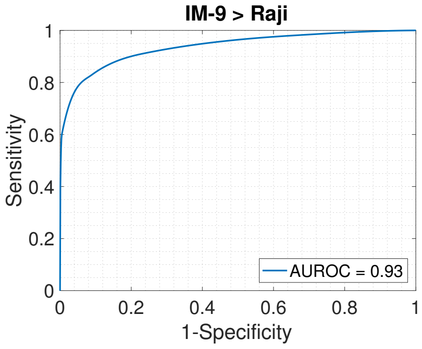

(d)

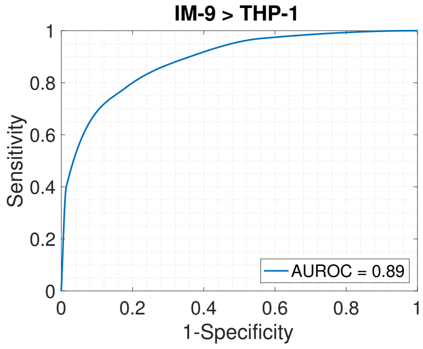

(e)

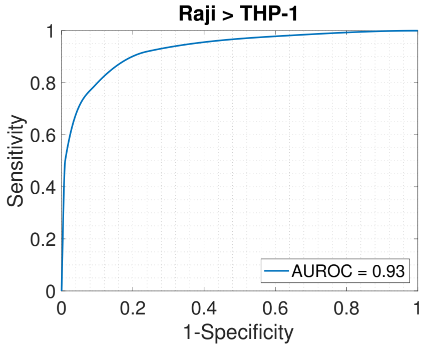

(f)

Fig B: Performance evaluation of the SMC method with the GSE11058 dataset. ROC plot is shown for all the cell types. For example, Jurkat > IM-9 implies that Jurkat is upregulated as compared to IM-9.

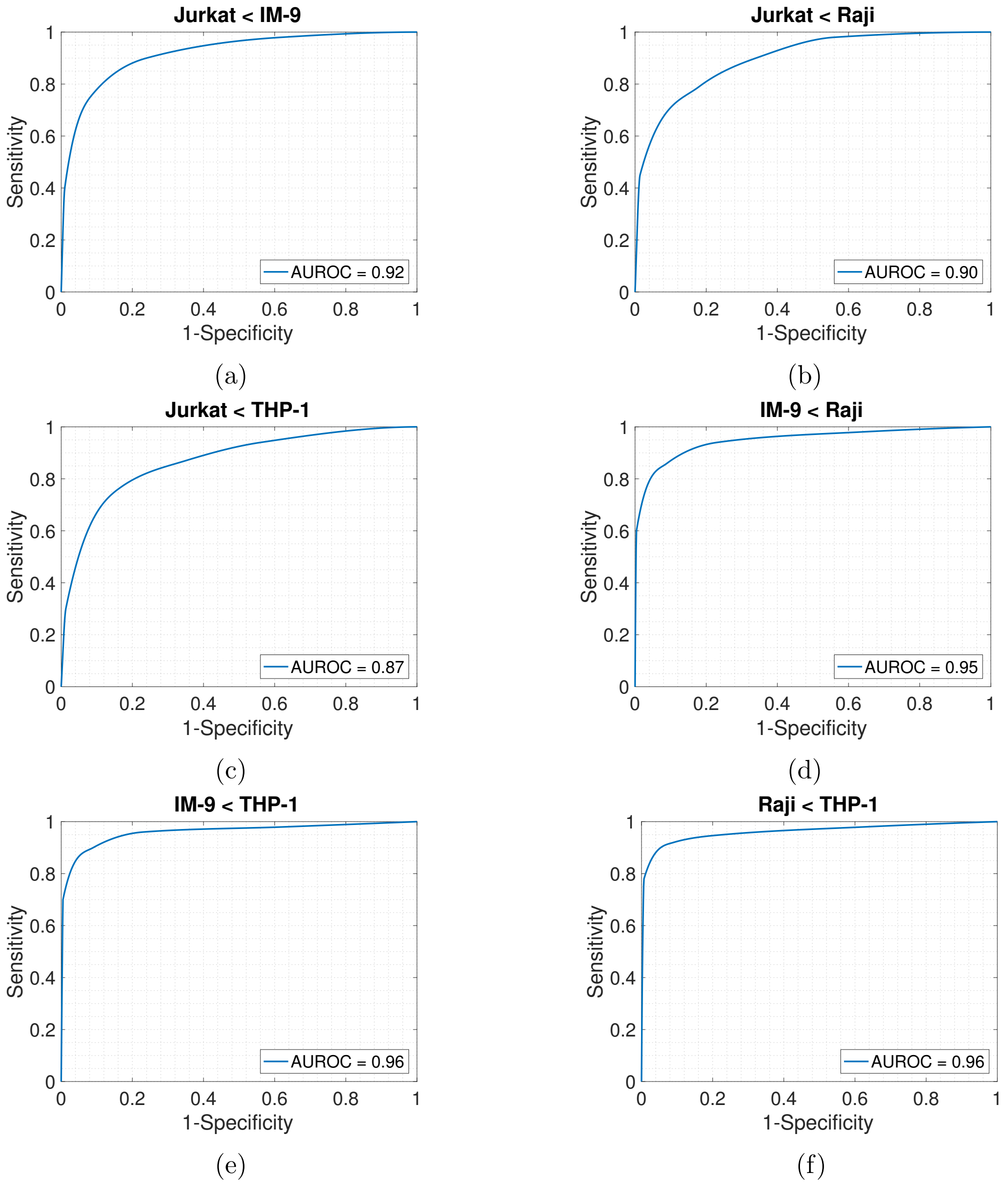

Fig C: Performance evaluation of the SMC method with the GSE11058 dataset. ROC plot is shown for all the cell types. For example, Jurkat $<$ IM-9 implies that Jurkat is downregulated as compared to IM-9.
